# Re-scaling and small area estimation of behavioral risk survey guided by social vulnerability data

**DOI:** 10.1186/s12889-022-14970-4

**Published:** 2023-01-27

**Authors:** Shaina L. Stacy, Hukum Chandra, Saurav Guha, Raanan Gurewitsch, Lu Ann L. Brink, Linda B. Robertson, David O. Wilson, Jian-Min Yuan, Saumyadipta Pyne

**Affiliations:** 1 UPMC Hillman Cancer Center, Pittsburgh, PA USA; 2 Department of Epidemiology, Graduate School of Public Health, University of Pittsburgh, Pittsburgh, PA USA; 3 ICAR-Indian Agricultural Statistics Research Institute, New Delhi, India; 4 Health Analytics Network, Pittsburgh, PA USA; 5 Public Health Dynamics Lab, Graduate School of Public Health, University of Pittsburgh, Pittsburgh, PA USA; 6 Allegheny County Health Department, Pittsburgh, PA USA; 7 Department of Medicine, University of Pittsburgh, Pittsburgh, PA USA; 8 Department of Statistics and Applied Probability, University of California Santa Barbara, Santa Barbara, CA USA

**Keywords:** Behavioral risk factor, BRFSS, Smoking, Spatial microsimulation, Small Area Estimation

## Abstract

**Background:**

Local governments and other public health entities often need population health measures at the county or subcounty level for activities such as resource allocation and targeting public health interventions, among others. Information collected via national surveys alone cannot fill these needs. We propose a novel, two-step method for rescaling health survey data and creating small area estimates (SAEs) of smoking rates using a Behavioral Risk Factor Surveillance System survey administered in 2015 to participants living in Allegheny County, Pennsylvania, USA.

**Methods:**

The first step consisted of a spatial microsimulation to rescale location of survey respondents from zip codes to tracts based on census population distributions by age, sex, race, and education. The rescaling allowed us, in the second step, to utilize available census tract-specific ancillary data on social vulnerability for small area estimation of local health risk using an area-level version of a logistic linear mixed model. To demonstrate this new two-step algorithm, we estimated the ever-smoking rate for the census tracts of Allegheny County.

**Results:**

The ever-smoking rate was above 70% for two census tracts to the southeast of the city of Pittsburgh. Several tracts in the southern and eastern sections of Pittsburgh also had relatively high (> 65%) ever-smoking rates.

**Conclusions:**

These SAEs may be used in local public health efforts to target interventions and educational resources aimed at reducing cigarette smoking. Further, our new two-step methodology may be extended to small area estimation for other locations and health outcomes.

**Supplementary Information:**

The online version contains supplementary material available at 10.1186/s12889-022-14970-4.

## Introduction

Disaggregation of behavioral risk data to finer geographical scales can provide key insights into many public health challenges. Researchers have noted, for example, high prevalence of cigarette smoking has continued among specific subpopulations in the United States (U.S.), many of whom are known to be vulnerable [1]. While tobacco smoking has declined considerably in the U.S. over the past decades, an estimated 13.7% of U.S. adults still smoke cigarettes, and it is the leading cause of preventable disease, disability, and death [2]. Cigarette smoking has been linked to many cardiovascular and respiratory diseases, such as chronic obstructive pulmonary disease (COPD), and is the leading risk factor for lung cancer development [3, 4]. Smoking cessation reduces the risk for these adverse health outcomes and can add as much as a decade to life expectancy [2]. A systematic combination of routinely collected health survey data with measures of local vulnerability can identify the neighborhoods with high smoking rates to allow better targeting of smoking cessation interventions, as well as those who may be experiencing disparities in outcomes of such programs.

National health surveys, such as the Behavioral Risk Factor Surveillance System (BRFSS) [5], are crucial tools for monitoring population trends in smoking and other high-risk, health-related behaviors at the country or state level. However, local governments and other public health entities often need these population health measures at the county or subcounty level for activities such as resource allocation and targeting public health interventions, among others. National surveys alone cannot fill these needs, often due to limited coverage of small geographic areas. Further, small sample sizes of such surveys when restricted to local populations make estimation of the variables of interest difficult, and possibly also unreliable below the state level. To address this issue, various small area estimation techniques have been proposed to downscale national or state health survey data and generate small area estimates (SAEs) that are deemed more reliable in terms of providing insights into health conditions and health-related risk behaviors that are specific to local populations [6]. A handful of prior studies have sought to produce SAEs based on BRFSS data, including for risk behaviors like smoking [7–11], health outcomes like COPD [12, 13], and other factors [14, 15].

In this study, our objective is to address the problem of estimating subcounty level behavioral risks, such as smoking rates, which can leverage on auxiliary data that generally exist for local populations but not necessarily on the desired spatial scale. Towards this, we introduced a new two-step algorithm for survey data to rescale and generate SAEs of the variable of interest. The term “small area” is used to describe a domain for which the sample size is not large enough to allow sufficiently precise direct survey estimation. Often indirect SAE methods depend on the availability of population level auxiliary information related to the variable of interest [6]. In the first step, we use microsimulation for spatial “side-scaling” of the survey data from the original unit of area (e.g., at zip code-level) to a different unit of area (e.g., at census tract-level). While uncertainty in this step may lead to loss of some data points, it can make valuable auxiliary information in the form of social vulnerability data available at this re-scaled level. In the second step, such local population level auxiliary data are used to inform the model for small area estimation, which, in this study, is done for every census tract (or simply “tract”). It also helps us avoid the use of zip codes of locations that may (and often do) change over time. Further, we include additional steps to decide whether to incorporate the design of the survey in our model and provide multiple model diagnostics. We demonstrated the methodology by estimating the tract level ever-smoking rates of Allegheny County in western Pennsylvania.

## Data and Methods

The University of Pittsburgh Institutional Review Board approved this study (STUDY19040081).

### Local BRFSS survey

The Allegheny County Health Department modeled its local BRFSS survey after the national survey, but the county raised its own funds for the survey and added many of its own questions. This county survey was administered via telephone to a random sample of adults 18 years and older who resided in Allegheny County in 2015. These methods have been described previously [16]; briefly, a probability-based sampling via random digital dial was conducted within the universes of all possible landline and cellular telephone numbers, 1.4 and 1.8 million total possible numbers, respectively. Six percent of possible landline and 4% of cellular telephone numbers in the county were sampled, with a total of 9032 interviews secured. Consent for participation was obtained at the beginning of the call [16]. For the present study, we obtained these as de-identified data, with personal identifying information masked by codes. We excluded 74 survey respondents with likely erroneous ages (< 18 years old) and 122 respondents with missing zip codes, leaving 8836 respondents in 105 zip code-defined areas for the spatial microsimulation. Survey demographic variables were re-categorized as necessary to harmonize with key census variables: sex (male or female), age (18–24, 25–34, 35–44, 45–64, ≥ 65 years), race (white, black, other), and education (less than high school, high school graduate, college 1 to 3 years (some college or technical school), and college graduate or higher). The sociodemographic profiles of the survey respondents are summarized in the Supplementary Table S1. For the present study, a respondent’s ever-smoking of tobacco in the form of cigarettes (not including e-cigarettes or further categories) data was used. For further details, see [16].

### American community survey

The spatial microsimulation (Step 1 below) requires census population margins by demographic factors to assign survey respondents to probable tracts. While the National Census takes place once every 10 years, the ACS is a nationwide survey that collects economic, housing, and demographic data every year [17]. We obtained 2015 tract-level population estimates from ACS to correspond to the year of our BRFSS survey.

### Social vulnerability data

The U.S. Centers for Disease Control and Prevention’s (CDC) Social Vulnerability Index (SVI) was originally computed to help public health officials and emergency response planners identify the most vulnerable communities that will require support during a hazardous event. The SVI ranks tracts on 15 social factors and further pools them into four summary themes: socioeconomic, household composition and disability, minority status and language, and housing type and transportation. It also provides an overall SVI [18].

### Spatial microsimulation

Step 1 of our two-step algorithm is a microsimulation to assign survey respondents to tracts using the approach of combinatorial optimization (CO). Details regarding this step are available in the Supplementary Methods. In short, this procedure involves the selection of an optimal combination of households from an existing survey dataset that best fit published small-area census tabulations [19]. We conducted the spatial microsimulation using the simPop package in R (version 4.0.2), an open-source data synthesizer that can be used to allocate populations from larger to smaller geographic areas [20]. Survey respondents were rescaled from zip codes to tracts based on census population marginals by age, sex, race, and education.

For CO based spatial microsimulation, we used the simPop package in R (version 4.0.2), an open-source data synthesizer that can be used to allocate populations from larger to smaller geographic areas [20]. After the study population was initially distributed to census tracts, a post-calibration procedure (calibPop) was performed to refine the distribution to tracts based on known census population marginals for age, sex, race, and education. This procedure implements CO based on simulated annealing to conduct an iterative search for a near optimal combination of households to populate the geographic areas. As this is a probabilistic step, a degree of randomness is involved in the household selection and the results will be slightly different for each run. Thus, the microsimulation was run for *N* = 100 iterations for each respondent *r*. In each iteration, *r* is assigned to at most one tract within her zip code that is known from the BRFSS survey data. Further, one census table containing a population breakdown by all four demographic variables of interest was not available. We therefore repeated the microsimulation for each of the following three combinations of marginals: {age, sex, race}; {age, sex, education}; and {sex, race, education}.

Then, we spatially assign to each respondent *r* the tract which has (i) the strongest assignment among (ii) the least inconsistent of all tracts assigned to *r* by microsimulation. Let *Max*(*r*, *d*) and *Min*(*r*, *d*) be the largest and the smallest number of assignments of any tract *d* to *r* out of a total of *N* = 100 microsimulations of *r* for each of the three combinations of marginals as stated above. For each *r*, we sort the tracts in a sequence {*d*_(*i*)_}_*r*_ in the increasing order of *Incons*(*r*, *d*_*j*_) =  *Max* (*r*, *d*_*j*_) −  *Min* (*r*, *d*_*j*_) as long as *Incons*(*r*, *d*_*j*_) < *δ*. Then *r* is assigned to the first tract in the sorted sequence {*d*_(*i*)_}_*r*_ for which *Max *(*r*, *d*_(*i*)_) ≥*μ*. The threshold values of *μ* and *δ* were selected as 40 and 50 based on the empirical distributions of *Max* and *Incons* to include a majority of respondents in the final assignments. If no tract met these criteria for a survey respondent, then that person was considered “unassigned” and excluded from Step 2.

### Small area estimation

In Step 2, we used the rescaled microdata from Step 1 for small area estimation of ever-smoking rates for all tracts in Allegheny County. Two types of variables are used for SAE analysis. First, the variable of interest drawn from the survey, i.e., ever-smoking, which is binary at the individual level, and corresponds to whether a person had ever smoked or not. The parameter of interest was to estimate the proportion of ever smokers within each census tract (given by the 458 tracts of Allegheny County).

The second type consists of the tract-level auxiliary variables (or covariates). We used as available covariates four theme-wise summary SVI variables defined as (i) Socioeconomic: RPL_THEME1, (ii) Household Composition & Disability: RPL_THEME2, (iii) Minority Status & Language: RPL_THEME3, and (iv) Housing Type & Transportation: RPL_THEME4. These values are given as percentile ranking.

A generalized linear model between tract-specific sample (unweighted) proportions of smoking and the set of four auxiliary variables (RPL_THEME1–4) was fitted for choosing the appropriate auxiliary variables. This model was fitted using the glm function in R and specifying the family as “binomial” and the tract-specific sample size as the weight. The primary purpose was to build a good explanatory and predictive model based on the available auxiliary data. Finally, two auxiliary variables, RPL_THEME1 (Socioeconomic) and RPL_THEME3 (Minority Status & Language), which significantly explained the model, were identified for use in subsequent SAE analysis.

The final model, including the covariates RPL_THEME1 and 3, was then used to produce tract-level estimates of ever-smoking rates. The tract-specific direct survey estimates of smoking rates were defined as follows. Let *y*_*di*_ denote the variable of interest for person *i* in tract *d* (*d* = 1, …*D*). In particular, *y*_*di*_ is a binary variable that takes the value 1 if person *i* in tract *d* smokes and 0 otherwise. Here, *D* is the total number of tracts in the study population, where *D*_1_ and *D*_2_ are the number of tracts with and without sample data, respectively, such that *D*_1_ + *D*_2_ = *D*. The aim is to estimate the proportion of ever smokers, ${P}_d={N}_d^{-1}{\sum}_{i=1}^{N_d}{y}_{di}$, in tract *d,* where *N*_*d*_ is the population size of tract *d.* Let *w*_*di*_ be the survey weight for person *i* in tract *d*. The direct estimator (denoted by *Direct*) for *P*_*d*_ is ${\hat{p}}_d^{Direct}={\left({\sum}_{i=1}^{n_d}{w}_{di}\right)}^{-1}{\sum}_{i=1}^{n_d}{w}_{di}{y}_{di}$, with the estimate of variance of the *Direct* estimator given by $v\left({\hat{p}}_d^{Direct}\right)\approx {\left({\sum}_{i=1}^{n_d}{w}_{di}\right)}^{-2}{\sum}_{i=1}^{n_d}{w}_{di}\left({w}_{di}-1\right){\left({y}_{di}-{\hat{p}}_d^{Direct}\right)}^2$, where *n*_*d*_ is sample size for tract *d.*

In case of simple random sampling (SRS) used for survey data collection, ${\hat{p}}_d^{Direct}={p}_d={\left({n}_d\right)}^{-1}{\sum}_{i=1}^{n_d}{y}_{di}$ is the simple sample proportion and $v\left({\hat{p}}_d^{Direct}\right)\approx {\left({n}_d\right)}^{-1}{p}_d\left(1-{p}_d\right)$, where $y={\sum}_{i=1}^{n_d}{y}_{di}$ denotes the sample count in tract *d*. If the sampling design is informative, this SRS-based version of *Direct* may be biased.

Let *u*_*d*_ denote the tract-specific random effects that capture the dissimilarities between the tracts. If we ignore the sampling design, the sample count *y*_*d*_ in tract *d* can be assumed to follow a binomial distribution with parameters *n*_*d*_ and *π*_*d*_, i.e., *y*_*d*_|*u*_*d*_ ∼ *Bin*(*n*_*d*_, *π*_*d*_); *d* = 1, …, *D*_1_. This leads to *E*(*y*_*d*_|*u*_*d*_) = *n*_*d*_*π*_*d*_. Let ***x***_*d*_ be the *k*-vector of covariates for tract *d* available from secondary data sources*.* Following previous work by study team members [21, 22] as well as others [23–25], the aggregate level version of logistic linear mixed model (LLMM) linking the probability *π*_*d*_ with the covariates ***x***_*d*_ is expressed as

1 $$logit\left({\pi}_d\right)=\mathit{\ln}\left\{{\pi}_d{\left(1-{\pi}_d\right)}^{-1}\right\}={\eta}_d={\boldsymbol{x}}_d^T\boldsymbol{\beta} +{u}_d$$

with ${\pi}_d=\mathit{\exp}\left({\boldsymbol{x}}_d^T\boldsymbol{\beta} +{u}_d\right){\left\{1+\mathit{\exp}\left({\boldsymbol{x}}_d^T\boldsymbol{\beta} +{u}_d\right)\right\}}^{-1}$. Here ***β*** is the *k*-vector of regression coefficients and *u*_*d*_ is assumed to be independent and normally distributed with mean zero and variance ${\sigma}_u^2$.

Assuming *N*_*d*_ >  >  > *n*_*d*_, an empirical plug-in predictor (EPP) of smoking proportion in tract *d* is given by

2 $${\hat{y}}_d^{EPP}=\mathit{\exp}\left({\boldsymbol{x}}_d^T\hat{\boldsymbol{\beta}}+{\hat{u}}_d\right){\left\{1+\mathit{\exp}\left({\boldsymbol{x}}_d^T\hat{\boldsymbol{\beta}}+{\hat{u}}_d\right)\right\}}^{-1};d=1,\dots, {D}_1$$

It is obvious that in order to compute the small area estimates by eq. (2), the estimates of the unknown parameters ***β*** and $\boldsymbol{u}={\left({u}_1,\dots, {u}_{D_1}\right)}^T$ in eq. (2) are obtained using an iterative procedure that combines the Penalized Quasi-Likelihood estimation of ***β*** and ***u*** with restricted maximum likelihood (REML) estimation of ${\sigma}_u^2$ to estimate unknown parameters. For tracts with no sample data (*n*_*d*_ = 0), the synthetic type predictor of smoking proportion in tract *d* is given by

3 $${\hat{y}}_d^{Syn}=\mathit{\exp}\left({\boldsymbol{x}}_d^T\hat{\boldsymbol{\beta}}\right){\left\{1+\mathit{\exp}\left({\boldsymbol{x}}_d^T\hat{\boldsymbol{\beta}}\right)\right\}}^{-1};d={D}_1+1,\dots, D$$

The mean squared error (MSE) estimation of small area predictor (2) and (3) is due to Chandra et al. (2019) [21].

To determine whether the sampling design used in survey data collection must be incorporated for valid inference about the population, we compute the effective sample sizes and the effective sample counts for the sample data, as described previously [21]. Use of effective sample size rather than the actual sample size allows for the varying information in each area under complex sampling. Following previous work, we use the effective sample sizes in place of observed sample sizes to incorporate the sampling design [22, 26].

### Diagnostic measures

These measures are used for examining the assumptions of the underlying models and assessing the empirical performances of the EPP method. Generally, two types of such measures are suggested and commonly employed in SAE application; (i) the model diagnostics, and (ii) the diagnostics for the small area estimates. The main purpose of model diagnostics is to verify the distributional assumptions of the underlying small area model, i.e., how well this working model performs when it is fitted to the survey data. The other diagnostics are used to validate reliability of the model-based small area estimates.

In LLMM, eq. (1), the random tract-specific effects are assumed to have a normal distribution with mean zero and fixed variance. If the model assumptions are satisfied, then the tract level random effects (or residuals) are expected to be randomly distributed and not significantly different from the regression line *y = 0;* whereas, from eq. (1) the area level random effects (or residuals) are defined as ${\hat{u}}_d={\hat{\eta}}_d-{\boldsymbol{x}}_d^T\hat{\boldsymbol{\beta}}d=1,\dots, D$. To examine the normality assumption, (as shown in Supplementary Fig. F1) the histogram (left plot), the normal probability (q-q) plot (center plot) and the distribution of the tract-level residuals (right plot) are used. The Shapiro-Wilk test (implemented using the shapiro.test() function in R) was also used to examine the normality of the tract random effects. The value of the Shapiro-Wilk test statistic was 0.984 with 285 degrees of freedom (*p*-value = 0.002). This indicates that the tract random effects are likely to be normally distributed. The tract level residuals appear to be randomly distributed around zero. Further, the histogram and q-q plot also provide evidence in support of the normality assumption (Supplementary Fig. F1).

Further, a set of diagnostics described previously [27, 28] are also considered for assessing validity and reliability of the tract-wise estimates generated by the EPP method. Here, we used 4 commonly used measures that address these requirements: a bias diagnostic, a goodness of fit test, a percent coefficient of variation diagnostic, and a 95% confidence interval diagnostic. The first two diagnostics examine the validity and last two assess the reliability or improved precision of the model-based small area estimates. In addition, we implemented a calibration diagnostic where the model-based estimates are aggregated to higher level and compared with direct survey estimates at this level. Here direct estimates DIR $\left({\hat{p}}_d^{Direct}\right)$ are defined as the survey weighted direct estimates. We compute bias (Bias) and average relative difference (RE) between direct $\left({\hat{p}}_d^{Direct}\right)$ and the EPP $\left({\hat{p}}_d^{EPP}\right)$ estimates as: $Bias={D}_1^{-1}{\sum}_{d=1}^{D_1}{\hat{p}}_d^{Direct}-{D}_1^{-1}\left({\sum}_{d=1}^{D_1}{\hat{p}}_d^{EPP}\right)$, and $RE={D}_1^{-1}{\sum}_{d=1}^{D_1}\left(\frac{{\hat{p}}_d^{Direct}-{\hat{p}}_d^{EPP}}{{\hat{p}}_d^{Direct}}\right)$ respectively. The calculated Bias is due to our model-based estimation step and not the randomization used in Step 1.

## Results

Out of the 8836 survey respondents used for the microsimulation in Step 1, 5901 (i.e., more than two-thirds) received a final tract assignment (Supplementary Fig. F2). In general, proportions of groups by education, race, and sex across the five age categories were similar between the 2015 census and our microsimulated datasets (Supplementary Fig. F3). Out of a total of 468 Allegheny County tracts in the survey data, we had 286 tracts with samples, and the rest were out of sample. In the sample data, the sample count (i.e., number of ever-smokers in the sample) was 4517. For this study, auxiliary variables were available for 458 tracts (285 with and 173 without sample data) only. Therefore, further analysis considered only 458 tracts for estimating the ever-smoking rate using SAE. At this stage, the survey data had a total sample size of 5892 respondents and sample count of 2689 (Table 1).

**Table 1:** Summary of sample size and sample count in survey data

Characteristics	Minimum	Maximum	Average	Total
Sample size	1	160	21	5892
Sample count (smoking incidence)	0	71	9	2689
Sampling fraction	0.0028	0.056	0.0092	

**Fig. 1:**
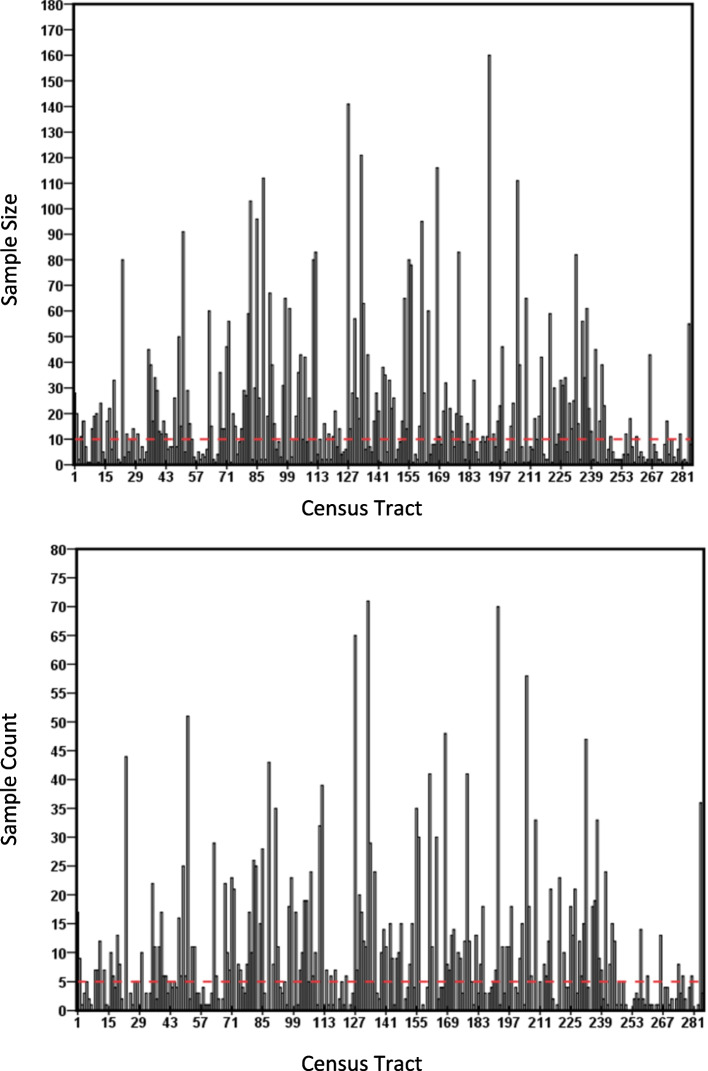
Tract-wise distribution of sample size (top) and sample count (bottom). The thresholds for sample size of 10 and sample count of 5 are shown with red horizontal lines

Across tracts, the sample size ranged from one to 160 with an average of 21. The average sample count was nine per tract, with a range of zero to 71. About 32% (91 out of 285) of total tracts had samples of less than five people (Fig. 1). As majority of the points are on the right side of the diagonal line (Supplementary Fig. F4a), it implies that for most tracts, the effective sample size is smaller than the observed sample size. Similarly, in most of the cases, the effective sample counts are smaller than the observed sample counts (Supplementary Fig. F4b). It is evident from the Supplementary Fig. F5 that the unweighted direct estimates underestimate the number of ever-smokers, which indicates that the sampling design is indeed informative, when compared to simple random sampling (SRS), in such tracts. Hence, the sampling weights cannot be ignored in our SAE analysis (Table 1).

We fitted generalized linear models between unweighted proportions of smoking and the four SVI themes to choose the appropriate auxiliary variables. The two auxiliary variables RPL_THEME1 and RPL_THEME3 were significant predictors for the ever-smoking rate with an Akaike Information Criterion (AIC) value of 1205.5 (Table 2). Further, the effects of ever-smoking were positive for RPL_THEME1 (coefficient: 0.824, *p* < 0.001) and negative for RPL_THEME3 (coefficient: − 0.633, *p* < 0.001). The null deviance of the model was 532 with 284 degrees of freedom, but adding RPL_THEME1 and RPL_THEME3 in the model reduced the residual deviance to 478 with a loss of two degrees of freedom. Using these covariates, the tract-level SAEs, and the corresponding standard errors, were computed. The results are shown in the Supplementary Table S2, along with census tract-specific socio-demographic variables described in the Supplementary Table S3. The excluded tracts are shown in the Supplementary Table S4.

**Table 2:** Model Parameters for the Generalised Linear Models for Smoking Rate. (^*^ *p* < 0.05; ^**^ *p* < 0.01; ^***^ *p* < 0.001)

Parameters	Estimate	Standard Error	z value	Pr(>|z|)
Intercept	−0.13177	0.06153	−2.142	0.0322 *
RPL_THEME1	0.82368	0.11753	7.008	2.42e-12 ***
RPL_THEME3	−0.63327	0.13015	−4.866	1.14e-06 ***
AIC	1205.5			
Null deviance	532.50 with 284 df			
Residual deviance	477.55 with 282 df			

*DF* degrees of freedom

**Fig. 2:**
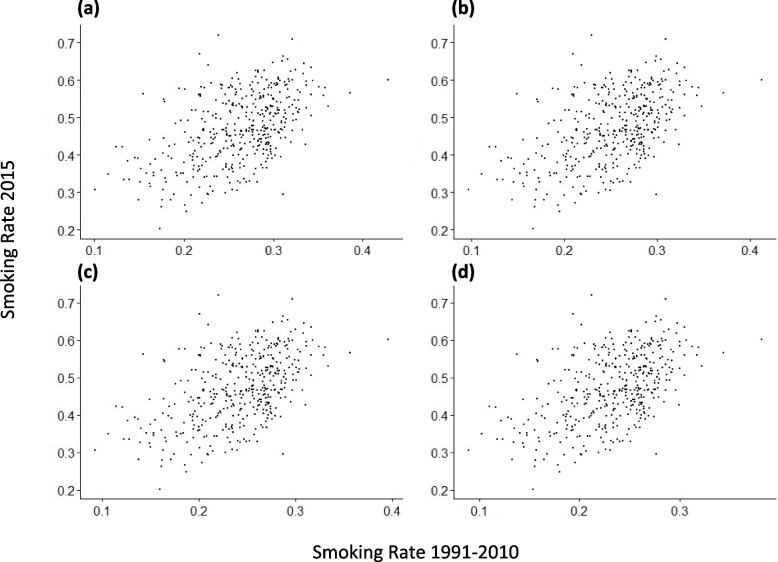
Scatterplots of SAEs of smoking rates calculated for 2015 (y-axis) in this study versus SAEs due to Oretega et al. (x-axis) for the years: (**a**) 1991–1995, (**b**) 1996–2000, (**c**) 2001–2005, and (d) 2006–2010

**Fig. 3:**
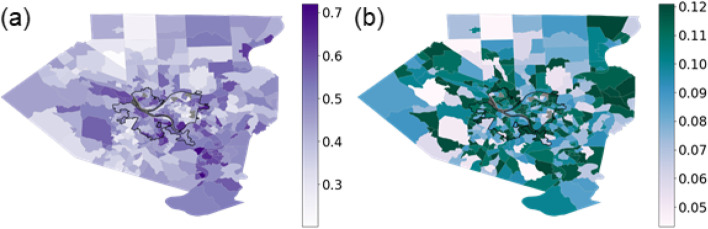
The maps show (**a**) the small area estimates and (**b**) standard errors (SE) of smoking rates in each tract of Allegheny County. The bold, black outline delineates the city of Pittsburgh

To validate our results, we compared our tract-level SAEs of ever-smoking rates with such estimates by a previous study [8] for the groups of years 1991–1995, 1996–2000, 2001–2005, and 2006–2010. Interestingly, the studies showed positive, significant correlations (correlation coefficients: ~ 0.51, *p* < 0.001) (Fig. 2). However, our rate estimates ranged from 20 to 72%, whereas these prior estimates had a narrower spread (~ 10–40%). In our analysis, the tracts with the highest estimated ever-smoking rate, slightly over 70%, were located southeast of the city of Pittsburgh. Other tracts with relatively high rates (> 65%) were located within neighborhoods in the southern (Hazelwood, Arlington, Carrick) and eastern (East Hills) sections. There was also a cluster of tracts with relatively high rates to the west of Pittsburgh (Fig. 3a). As expected, the standard errors of SAE are higher in non-sample tracts (Fig. 3b). Distributions were similar between tracts in the city of Pittsburgh versus outside of Pittsburgh, although the SAEs for non-city tracts had slightly more spread (Supplementary Fig. F6).

## Discussion

In this study, we have developed a new two-step algorithm for rescaling behavioral survey data and modeling the prevalence of small area-level behaviors. Health surveys, including the BRFSS and others, often do not provide spatial resolution below the state or county level. The local BRFSS survey administered in Allegheny County did collect zip code of residence, but without tract assignments, linkage with informative, ancillary data sources, such as the SVI, is difficult. Our microsimulation step allowed us to distribute survey respondents to tracts within the study area in a way that reflected the known sociodemographic composition of the tracts. While not every survey respondent received a final tract assignment, we gained in spatial resolution for the others. Such disaggregation of population data at an informative spatial scale can mitigate statistical bias that may appear in the form of modifiable areal unit problem (MAUP) [29].

According to the most recent Surgeon General’s report, 13.7% of U.S. adults smoke [2]. Although the adult smoking rate in Allegheny County decreased from 23% in 2009–2010 to 19% in 2017 [2], this still exceeds the national rate. Racial disparities also persist in the county, both for smoking and smoking-related health outcomes. African Americans are both more likely to smoke (30% versus 17% of whites) and have rates of lung cancer 15–30% higher than whites [14]. The SAEs of smoking rates demonstrated in this study, and its rigorous use of tract-specific (socioeconomic, and minority & language-based) vulnerability covariates in the estimation, could inform local smoking cessation interventions to further decrease smoking rates in the county, particularly for high-risk groups such as those with higher levels of poverty or unemployment. In addition, lower socioeconomic communities face greater burdens of environmental pollution [30], further compounding their risks for cancer and other diseases.

Past applications of SAE on BRFSS data, e.g., Zhang et al. (2014) [12], were based on fitting a unit level logistic linear mixed model to BRFSS data and then drawing 1000 random samples from their estimated conditional distributions using the fitted model parameters, and thus, generating a sample of 1000 SAEs for each small area defined in the study [10]. The efficacy of the generated SAEs is therefore highly dependent upon the fitted model. The SAE method under an area level, logistic linear mixed model applied in this paper is a widely used approach if the model covariates are only available in aggregate form. It has a simple and closed form expression and, therefore, national statistical agencies (e.g., Office for National Statistics, Australian Bureau of Statistics, etc.) often prefer it.

Yet, the present study has some limitations. The spatial re-scaling in Step 1 to gain in terms of the ability to include insightful covariates has a potential cost in terms of some loss of samples. In the probabilistic CO procedure of Step 1, a degree of randomness is involved in the spatial assignments [19]. Given the current methodological limitation in terms of the ability for estimating uncertainty in the results of microsimulation, algorithms such as CO could introduce bias for the small area estimates. This is, however, a more general problem which needs to be addressed in future work. In Step 2, as one would expect, standard errors were higher among non-sample compared to sample tracts. Caution should be used in interpreting the SAE results in these non-sample tracts. We do not have reliable, direct-estimate data to validate our SAE census tract results, although they correlate significantly with those from past studies. Finally, while these tract-level estimates may be used to target smoking cessation interventions or help identify high-risk communities for smoking and related health outcomes, they cannot be used to draw inferences about smoking habits of specific individuals residing in the small areas.

## Conclusion

We proposed a two-step algorithm for rescaling survey data to more granular geographic levels for which ancillary data may be available to produce locally relevant estimates for health-related risk behaviors at these levels. We used smoking rates in Allegheny County, PA, both as a case study to demonstrate the algorithm as well as to create tract-level estimates that may be used in local public health interventions or additional studies. Future work could leverage on the methods described here for other health surveys, locations, diseases, and health-related behaviors.

## Supplementary Information

**Additional file 1: Fig. F1.** Histograms (left plot), normal q-q plots (center plot) and distributions of the tract level residuals (right plot). **Fig. F2.** Scatterplot of *Incons* (*Max-Min*) versus *Max* values for each of *N* =8836 survey respondents due to spatial assignments in three sets of 100 microsimulations. Empirically, the dotted lines show the most inclusive thresholds at *Max* ≥ 40 and *Incons* < 50. The resulting included assignments are shown as red dots. **Fig. F3.** Barplots comparing the 2015 Census data (C) and Microsimulation results (M) with paired bars that show the proportions of each category of (a) sex, (b) race, and (c) education across 5 groups of increasing age. **Fig. F4**. Tract-wise effective sample size vs. observed sample size (a), and effective sample count vs. observed sample count (b). **Fig. F5.** Tract-wise survey weighted vs. unweighted direct estimates of smoking rates. **Fig. F6.** Box plots comparing small area estimates of smoking rates and standard errors between the city of Pittsburgh and non-Pittsburgh tracts. **Table S1.** The socio-demographic profiles of the BRFSS survey respondents (Allegheny County, PA, 2015–2016) are shown. The data are due to Table 8 in the report, ‘*Results from the 2015-2016 Allegheny County Health Survey: Measuring the Health of Adult Residents*’, K. Hacker, et al. (2017), Allegheny County Health Department, Pittsburgh, PA. (Reference 16, main paper.). **Table S2.** Tract-specific small area estimates and associated variables for Allegheny County, PA. **Table S3.** Data dictionary for the variables in Table S2. **Table S4.** Tracts removed from small area analysis.

## Data Availability

The datasets generated and analyzed during the current study are not publicly available but are available from the corresponding author on reasonable request.

## Abbreviations

ACS: American Community Survey
AIC: Akaike Information Criterion
BRFSS: Behavioral Risk Factor Surveillance System
CO: Combinatorial Optimization
COPD: Chronic Obstructive Pulmonary Disease
EPP: Empirical Plug-In Predictor
LLMM: Logistic Linear Mixed Model
MAUP: Modifiable Areal Unit Problem
MSE: Mean Squared Error
SVI: Social Vulnerability Index
U.S. E.P.A.: United States Environmental Protection Agency
