# Supplementary material for: Re-scaling and small area estimation of behavioral risk survey guided by social vulnerability data
Source: BMC Public Health. 2023 Jan 27;23:184. doi: 10.1186/s12889-022-14970-4 (PMC9881361; doi:10.1186/s12889-022-14970-4)
Supplement: Supplementary file 1 — Additional file 1: Fig. F1. Histograms (left plot), normal q-q plots (center plot) and distributions of the tract level residuals (right plot). Fig. F2. Scatterplot of Incons (Max-Min) versus Max values for each of N =8836 survey respondents due to spatial assignments in three sets of 100 microsimulations. Empirically, the dotted lines show the most inclusive thresholds at Max ≥ 40 and Incons < 50. The resulting included assignments are shown as red dots. Fig. F3. Barplots comparing the 2015 Census data (C) and Microsimulation results (M) with paired bars that show the proportions of each category of (a) sex, (b) race, and (c) education across 5 groups of increasing age. Fig. F4. Tract-wise effective sample size vs. observed sample size (a), and effective sample count vs. observed sample count (b). Fig. F5. Tract-wise survey weighted vs. unweighted direct estimates of smoking rates. Fig. F6. Box plots comparing small area estimates of smoking rates and standard errors between the city of Pittsburgh and non-Pittsburgh tracts. Table S1. The socio-demographic profiles of the BRFSS survey respondents (Allegheny County, PA, 2015–2016) are shown. The data are due to Table 8 in the report, ‘Results from the 2015-2016 Allegheny County Health Survey: Measuring the Health of Adult Residents’, K. Hacker, et al. (2017), Allegheny County Health Department, Pittsburgh, PA. (Reference 16, main paper.). Table S2. Tract-specific small area estimates and associated variables for Allegheny County, PA. Table S3. Data dictionary for the variables in Table S2. Table S4. Tracts removed from small area analysis. [file 12889_2022_14970_MOESM1_ESM.docx]

**Supplementary Material: Re-scaling and Small Area Estimation of Behavioral Risk Survey guided by Social Vulnerability Data**

Shaina L. Stacy, Hukum Chandra, Saurav Guha, Raanan Gurewitsch, LuAnn L. Brink,

Linda B. Robertson, David O. Wilson, Jian-Min Yuan, Saumyadipta Pyne


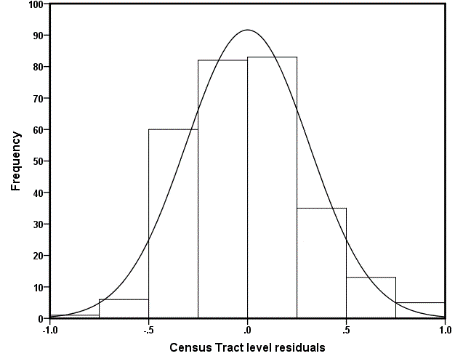

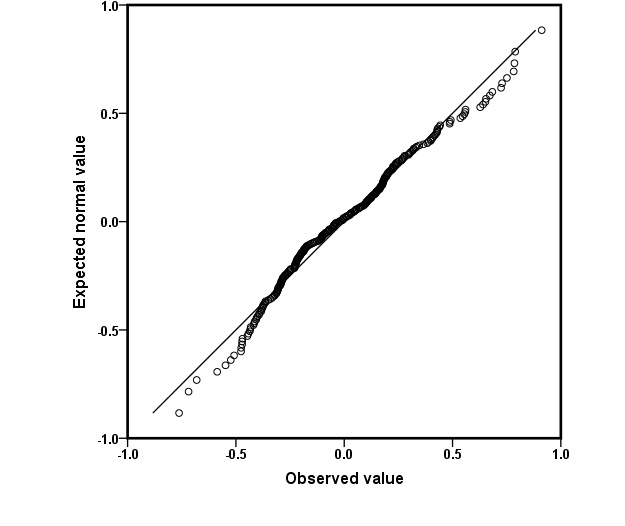

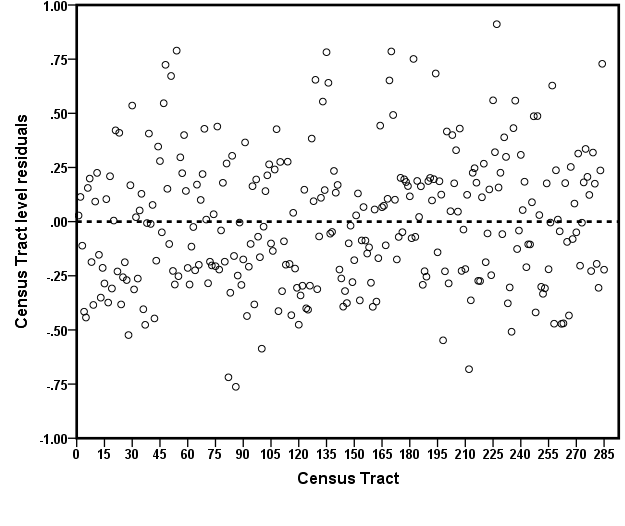
**Figure F1.** Histograms (left plot), normal q-q plots (center plot) and distributions of the tract level residuals (right plot).


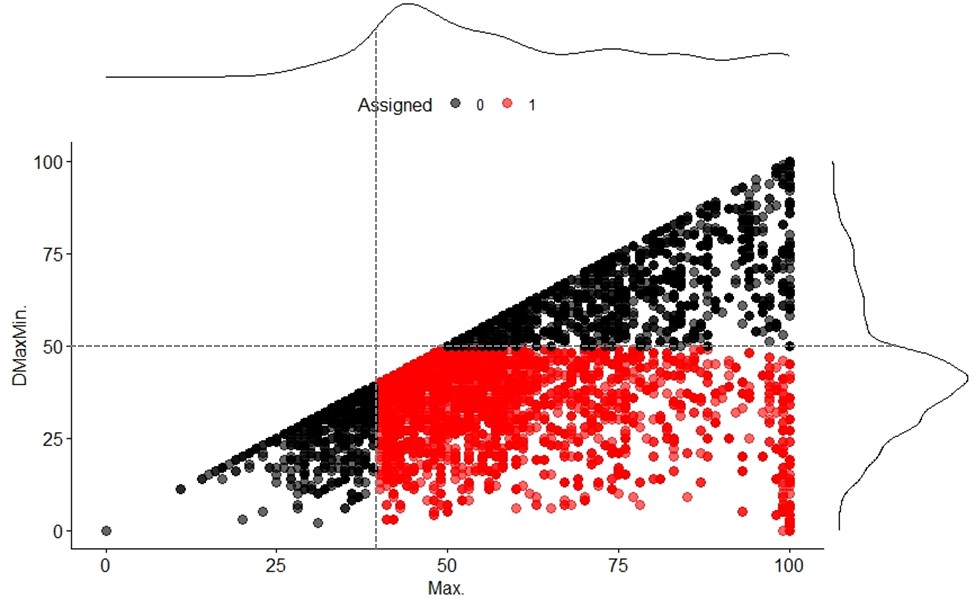


Max

Max-Min

**Figure F2.** Scatterplot of *Incons* (*Max-Min*) versus *Max* values for each of $N$=8836 survey respondents due to spatial assignments in three sets of 100 microsimulations. Empirically, the dotted lines show the most inclusive thresholds at *Max* ≥40 and *Incons* <50. The resulting included assignments are shown as red dots.


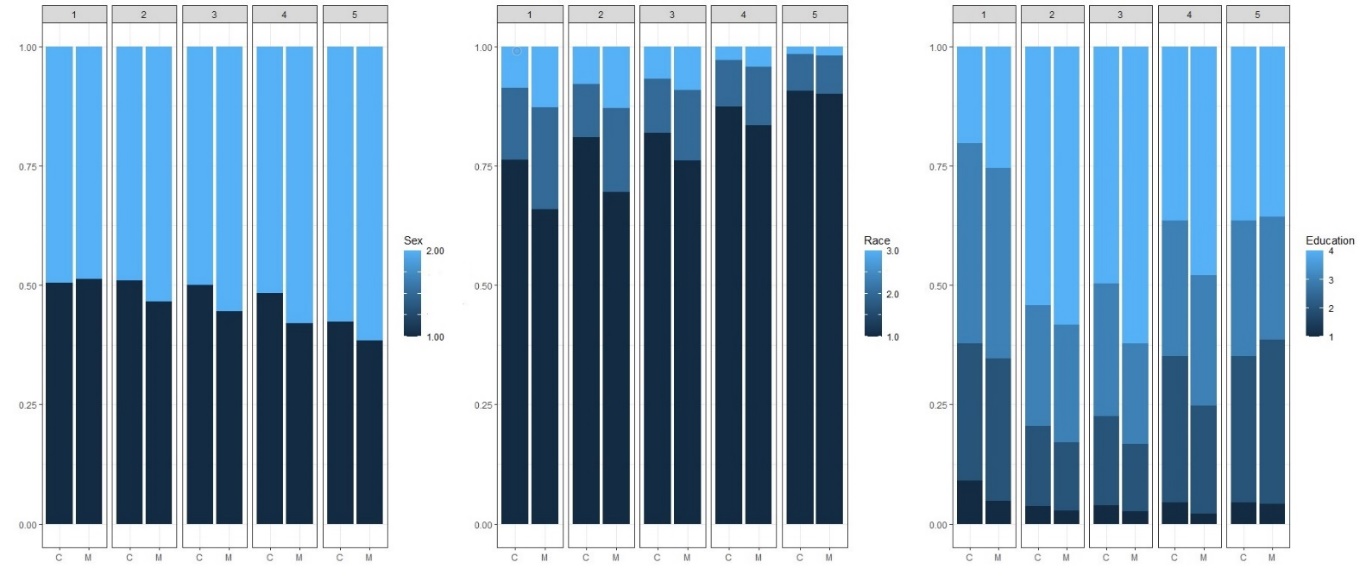


Dataset

Proportion

**(a)**

**(b)**

**(c)**

**Figure F3.** Barplots comparing the 2015 Census data (C) and Microsimulation results (M) with paired bars that show the proportions of each category of (a) sex, (b) race, and (c) education across 5 groups of increasing age.


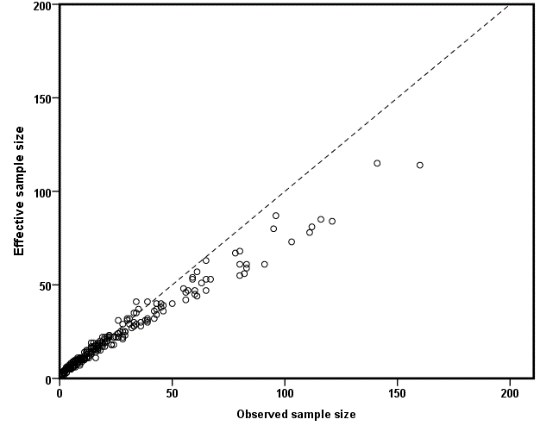

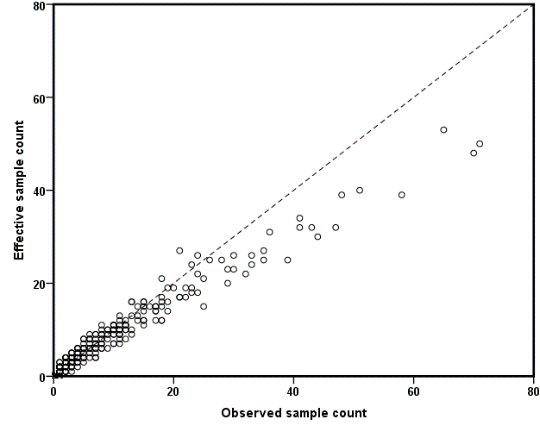


**(a) (b)**

**Figure F4.** Tract-wise effective sample size vs. observed sample size (a), and effective sample count vs. observed sample count (b).


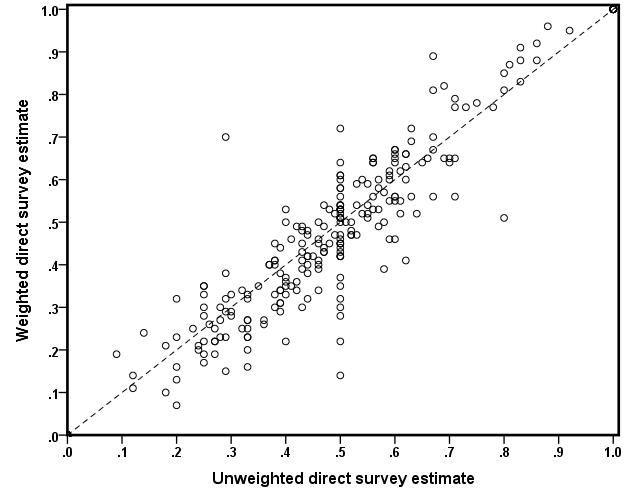


**Figure F5.** Tract-wise survey weighted vs. unweighted direct estimates of smoking rates.

**
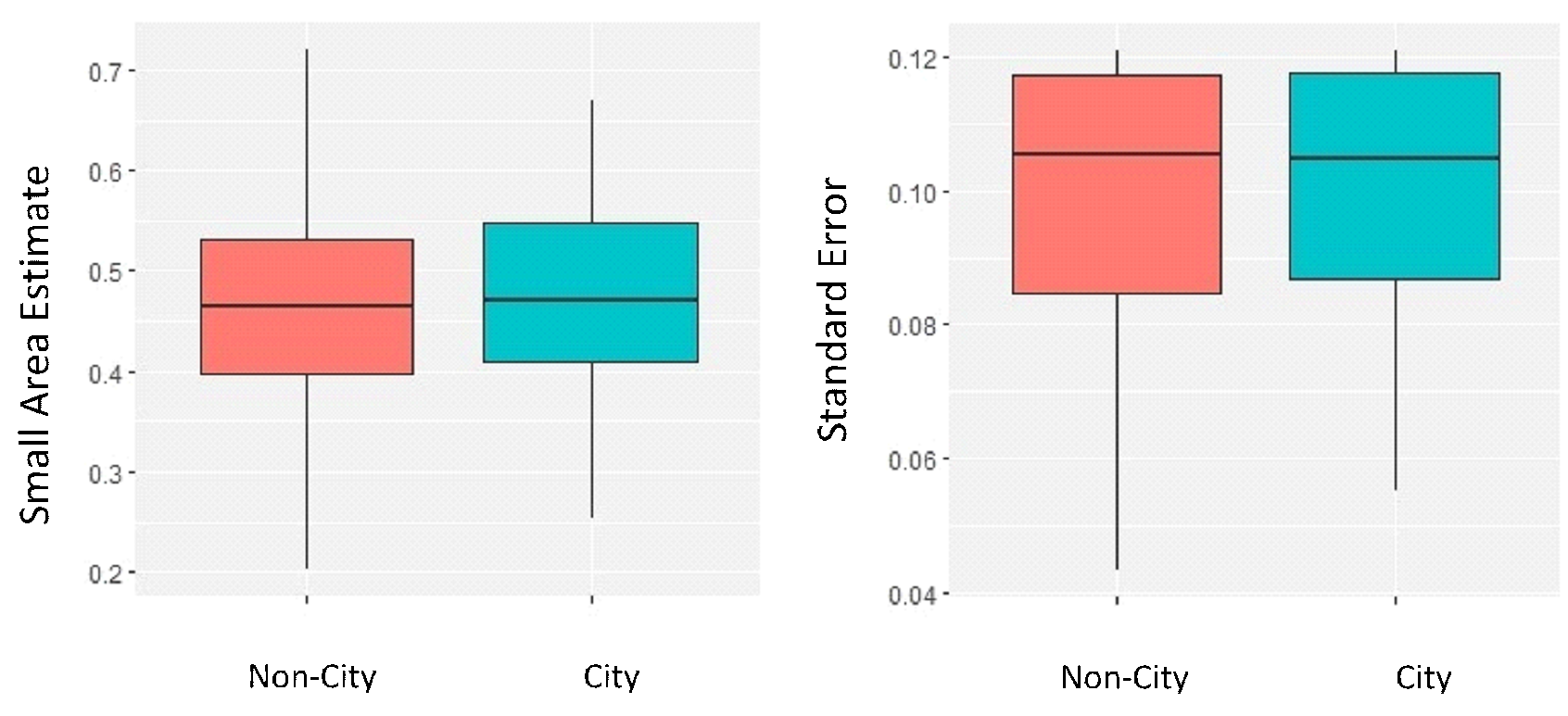
**

**Figure F6.** Box plots comparing small area estimates of smoking rates and standard errors between the city of Pittsburgh and non-Pittsburgh tracts.

**Table S1.** The socio-demographic profiles of the BRFSS survey respondents (Allegheny County, PA, 2015-2016) are shown. The data are due to Table 8 in the report, ‘*Results from the 2015-2016 Allegheny County Health Survey: Measuring the Health of Adult Residents*’, K. Hacker, et. al. (2017), Allegheny County Health Department, Pittsburgh, PA. (Reference 16, main paper.)

| Survey Respondents from Allegheny County, PA, 2015-2016 | | | | |
| --- | --- | --- | --- | --- |
|  | Total  No. of Adults | No. of Current Smokers | % | CI |
| All Adults | 8840 | 1447 | 19 | 18-20 |
| Male | 3722 | 624 | 19 | 18-21 |
| Female | 5118 | 823 | 19 | 17-20 |
| Age: |  |  |  |  |
| 18–29 | 960 | 187 | 20 | 18-23 |
| 30–44 | 1501 | 307 | 24 | 21-26 |
| 45–64 | 3548 | 679 | 22 | 20-23 |
| 65+ | 2762 | 264 | 10 | 8-11 |
| Education: |  |  |  |  |
| < High School | 261 | 98 | 44 | 37-51 |
| High School | 2172 | 514 | 27 | 25-29 |
| Some College | 2347 | 464 | 20 | 18-22 |
| College Degree | 4049 | 370 | 9 | 8-10 |
| Household Income |  |  |  |  |
| < $15,000 | 634 | 244 | 42 | 37-46 |
| $15,000–24,999 | 1053 | 268 | 29 | 26-33 |
| $25,000–49,999 | 1850 | 369 | 22 | 20-24 |
| $50,000–74,999 | 1301 | 193 | 17 | 14-19 |
| $75,000+ | 2942 | 257 | 10 | 9-12 |
| Race: |  |  |  |  |
| White | 7216 | 1081 | 17 | 16-19 |
| Black | 1059 | 275 | 30 | 26-34 |
| Other | 487 | 75 | 18 | 14-23 |

**Table S2.** Tract-specific small area estimates and associated variables for Allegheny County, PA

| **TRACT** | **Indicator** | **CX1** | **CX2** | **Small area estimates** | **SE** | **%CV** | **Lower** | **Upper** | **RPL_THEME1** | **RPL_THEME3** | **Adult_Percent** | **Poverty_Percent** | **Unemployed_Percent** | **No**  **HighSchool**  **Diploma_Percent** | **Minority_Percent** |
| --- | --- | --- | --- | --- | --- | --- | --- | --- | --- | --- | --- | --- | --- | --- | --- |
| 42003010300 | 1 | -79.98517112 | 40.4357381 | 0.566 | 0.08142 | 14.39 | 0.406 | 0.726 | 0.8951 | 0.6351 | 98.2 | 35.1 | 11.2 | 13.1 | 34.4 |
| 42003020100 | 1 | -80.00003326 | 40.4407124 | 0.405 | 0.07994 | 19.74 | 0.248 | 0.562 | 0.2657 | 0.7273 | 98 | 19.1 | 5.6 | 1.8 | 21.6 |
| 42003020300 | 0 | -79.98143199 | 40.45427401 | 0.336 | 0.10787 | 32.11 | 0.125 | 0.547 | 0.0351 | 0.6489 | 95.9 | 6 | 3.6 | 0.4 | 17.2 |
| 42003030500 | 1 | -79.98380174 | 40.44270925 | 0.492 | 0.11472 | 23.32 | 0.267 | 0.717 | 0.9321 | 0.8969 | 79.6 | 36.3 | 17.4 | 17.3 | 92.6 |
| 42003040200 | 0 | -79.96634777 | 40.44011037 | 0.551 | 0.11962 | 21.71 | 0.317 | 0.786 | 0.8961 | 0.6776 | 87.8 | 47.4 | 21.1 | 9.8 | 65.6 |
| 42003040400 | 1 | -79.94937061 | 40.44734717 | 0.343 | 0.08781 | 25.60 | 0.171 | 0.515 | 0.4651 | 0.6548 | 99.2 | 44.7 | 5.8 | 0.9 | 39.8 |
| 42003040500 | 1 | -79.95595388 | 40.43961945 | 0.464 | 0.08683 | 18.71 | 0.294 | 0.634 | 0.7127 | 0.2949 | 99.2 | 77.7 | 6 | 4.1 | 16.4 |
| 42003040600 | 1 | -79.95228578 | 40.43675045 | 0.556 | 0.10512 | 18.91 | 0.350 | 0.762 | 0.8466 | 0.7888 | 100 | 64.4 | 4.6 | 28.5 | 17.1 |
| 42003040900 | 1 | -79.95789639 | 40.43327584 | 0.575 | 0.11428 | 19.87 | 0.351 | 0.799 | 0.8479 | 0.746 | 93.9 | 55.2 | 9.8 | 9.9 | 24.1 |
| 42003050100 | 0 | -79.97289016 | 40.4477128 | 0.520 | 0.12069 | 23.19 | 0.284 | 0.757 | 0.9396 | 0.9028 | 79.6 | 35.6 | 20.6 | 19.2 | 91.9 |
| 42003050600 | 1 | -79.95979339 | 40.45317485 | 0.399 | 0.11207 | 28.09 | 0.179 | 0.619 | 0.6344 | 0.8803 | 88.6 | 21.7 | 9.5 | 8.8 | 80.6 |
| 42003050900 | 0 | -79.9743607 | 40.4510292 | 0.538 | 0.12019 | 22.34 | 0.302 | 0.773 | 0.9693 | 0.8507 | 66.3 | 68.1 | 31.5 | 15.2 | 100 |
| 42003051000 | 0 | -79.96753448 | 40.44438017 | 0.543 | 0.11998 | 22.09 | 0.308 | 0.778 | 0.8482 | 0.6529 | 91.3 | 54 | 7.6 | 10.5 | 57.5 |
| 42003051100 | 0 | -79.97646428 | 40.44165887 | 0.601 | 0.11591 | 19.27 | 0.374 | 0.829 | 0.9825 | 0.5245 | 78.1 | 36.9 | 41.4 | 34.1 | 100 |
| 42003060300 | 1 | -79.96505834 | 40.46525671 | 0.403 | 0.08803 | 21.84 | 0.230 | 0.576 | 0.4704 | 0.3633 | 85.4 | 18.3 | 7.9 | 6.3 | 26.5 |
| 42003060500 | 0 | -79.96624064 | 40.45763875 | 0.511 | 0.12083 | 23.65 | 0.274 | 0.748 | 0.4307 | 0.2396 | 94.1 | 14.9 | 5.8 | 7.4 | 12.1 |
| 42003070300 | 1 | -79.93520444 | 40.45078406 | 0.391 | 0.07823 | 20.01 | 0.238 | 0.544 | 0.1158 | 0.5676 | 88.3 | 11.8 | 1.3 | 4.4 | 16.4 |
| 42003070500 | 1 | -79.92896176 | 40.4560166 | 0.411 | 0.07937 | 19.31 | 0.255 | 0.567 | 0.2294 | 0.7938 | 95.2 | 24.1 | 3.7 | 0.6 | 29.8 |
| 42003070600 | 1 | -79.92547722 | 40.45327032 | 0.328 | 0.10286 | 31.36 | 0.126 | 0.530 | 0.1202 | 0.6151 | 95.2 | 13.9 | 1.9 | 0.5 | 21.7 |
| 42003070800 | 0 | -79.91976728 | 40.45647078 | 0.341 | 0.10864 | 31.87 | 0.128 | 0.554 | 0.226 | 0.8872 | 90.8 | 23.7 | 3.5 | 1 | 38.2 |
| 42003070900 | 1 | -79.94195091 | 40.45202928 | 0.295 | 0.07113 | 24.11 | 0.156 | 0.434 | 0.3421 | 0.8622 | 95.8 | 31.9 | 4.3 | 4.1 | 44.5 |
| 42003080200 | 1 | -79.9513354 | 40.45996593 | 0.383 | 0.10203 | 26.64 | 0.183 | 0.583 | 0.308 | 0.4777 | 91.8 | 18.5 | 2.8 | 4.3 | 12.2 |
| 42003080400 | 0 | -79.94590637 | 40.45633555 | 0.443 | 0.11931 | 26.93 | 0.209 | 0.677 | 0.3862 | 0.5398 | 97.7 | 31.2 | 3.7 | 0.3 | 20.8 |
| 42003080600 | 0 | -79.93966739 | 40.46019853 | 0.413 | 0.11724 | 28.37 | 0.183 | 0.643 | 0.3061 | 0.5892 | 87.1 | 11.2 | 7 | 2.5 | 9.2 |
| 42003080700 | 1 | -79.93437821 | 40.4609729 | 0.305 | 0.09560 | 31.35 | 0.118 | 0.492 | 0.3252 | 0.8619 | 91.2 | 25 | 2.5 | 1.8 | 34.1 |
| 42003080900 | 0 | -79.94424976 | 40.46298967 | 0.354 | 0.11065 | 31.21 | 0.138 | 0.571 | 0.2401 | 0.8272 | 89.7 | 15.7 | 1.1 | 5.2 | 24.4 |
| 42003090100 | 1 | -79.96197129 | 40.47530305 | 0.437 | 0.08832 | 20.21 | 0.264 | 0.610 | 0.3894 | 0.7142 | 95.4 | 14.1 | 5.1 | 9 | 14.9 |
| 42003090200 | 1 | -79.95714572 | 40.46889289 | 0.330 | 0.07314 | 22.16 | 0.187 | 0.473 | 0.3865 | 0.6785 | 90.5 | 12.3 | 4.6 | 8.4 | 14 |
| 42003090300 | 1 | -79.95294238 | 40.46435457 | 0.567 | 0.09788 | 17.26 | 0.375 | 0.759 | 0.7208 | 0.6236 | 91.5 | 15.5 | 11.7 | 8.1 | 17.3 |
| 42003100500 | 1 | -79.93698077 | 40.47713028 | 0.369 | 0.07183 | 19.47 | 0.228 | 0.510 | 0.5966 | 0.8354 | 79.3 | 18.6 | 6.5 | 9.8 | 73.7 |
| 42003101100 | 1 | -79.94975779 | 40.48388148 | 0.427 | 0.08939 | 20.93 | 0.252 | 0.602 | 0.4673 | 0.7457 | 83.6 | 14.7 | 4.7 | 7.8 | 26.9 |
| 42003101400 | 1 | -79.92708078 | 40.48514866 | 0.550 | 0.10886 | 19.79 | 0.337 | 0.763 | 0.4138 | 0.5692 | 81.5 | 11 | 4.5 | 9 | 16.8 |
| 42003101600 | 0 | -79.93933032 | 40.47037375 | 0.576 | 0.11810 | 20.51 | 0.344 | 0.807 | 0.8682 | 0.5042 | 60.5 | 45.5 | 19.1 | 13.9 | 96.5 |
| 42003101700 | 1 | -79.94106762 | 40.46687894 | 0.492 | 0.11619 | 23.62 | 0.264 | 0.720 | 0.7264 | 0.4517 | 85.3 | 42.2 | 9.4 | 3.2 | 66.5 |
| 42003101800 | 0 | -79.93693209 | 40.4841698 | 0.366 | 0.11223 | 30.65 | 0.146 | 0.586 | 0.1915 | 0.6917 | 83.3 | 5 | 3.7 | 7.3 | 24.3 |
| 42003110200 | 1 | -79.92316283 | 40.47555175 | 0.514 | 0.05933 | 11.54 | 0.398 | 0.630 | 0.1753 | 0.4127 | 79.2 | 11.7 | 3.5 | 1.5 | 42.4 |
| 42003110600 | 1 | -79.91559873 | 40.4726919 | 0.272 | 0.08626 | 31.71 | 0.103 | 0.441 | 0.1502 | 0.7017 | 76.7 | 11.9 | 2.2 | 3.8 | 18.3 |
| 42003111300 | 1 | -79.92449647 | 40.46898213 | 0.340 | 0.08258 | 24.29 | 0.178 | 0.502 | 0.4476 | 0.8625 | 81 | 17.1 | 3.9 | 9.7 | 61.5 |
| 42003111400 | 0 | -79.93184335 | 40.46701105 | 0.582 | 0.11764 | 20.21 | 0.351 | 0.813 | 0.8714 | 0.4758 | 78.3 | 33.3 | 16 | 11.5 | 83.9 |
| 42003111500 | 0 | -79.92463974 | 40.4622074 | 0.497 | 0.12088 | 24.34 | 0.260 | 0.734 | 0.8081 | 0.8441 | 81.8 | 29.4 | 11.5 | 8.9 | 76.1 |
| 42003120300 | 0 | -79.90292176 | 40.46939291 | 0.561 | 0.11907 | 21.21 | 0.328 | 0.795 | 0.8166 | 0.5111 | 77.8 | 27.4 | 24 | 7.6 | 97.7 |
| 42003120400 | 0 | -79.91045159 | 40.4653383 | 0.580 | 0.11782 | 20.33 | 0.349 | 0.811 | 0.8792 | 0.4995 | 76.7 | 44 | 33.3 | 8.4 | 95.2 |
| 42003120700 | 0 | -79.90394627 | 40.45943112 | 0.577 | 0.11805 | 20.48 | 0.345 | 0.808 | 0.8823 | 0.5208 | 83.1 | 17.3 | 11.5 | 20.3 | 99.4 |
| 42003120800 | 0 | -79.91313416 | 40.46037439 | 0.548 | 0.11977 | 21.85 | 0.313 | 0.783 | 0.9543 | 0.7751 | 75.7 | 24.2 | 40.6 | 31.5 | 79.6 |
| 42003130100 | 1 | -79.88868914 | 40.45925096 | 0.467 | 0.10691 | 22.89 | 0.257 | 0.677 | 0.964 | 0.9713 | 67.2 | 53.9 | 29.5 | 15.2 | 100 |
| 42003130200 | 1 | -79.89349488 | 40.45869395 | 0.515 | 0.09813 | 19.05 | 0.323 | 0.707 | 0.8939 | 0.5073 | 61.9 | 40.7 | 16.7 | 12.9 | 97 |
| 42003130300 | 1 | -79.89683284 | 40.45462781 | 0.446 | 0.09061 | 20.32 | 0.268 | 0.624 | 0.8707 | 0.5086 | 77.6 | 39.6 | 22.5 | 9.1 | 97 |
| 42003130400 | 1 | -79.88828734 | 40.45176745 | 0.641 | 0.10886 | 16.98 | 0.428 | 0.854 | 0.9718 | 0.508 | 72.3 | 40.4 | 20 | 23.4 | 97 |
| 42003130600 | 1 | -79.87527925 | 40.45386729 | 0.670 | 0.08444 | 12.60 | 0.504 | 0.836 | 0.8955 | 0.721 | 77.7 | 40.9 | 18.7 | 11.6 | 93.8 |
| 42003140100 | 1 | -79.93734375 | 40.44274256 | 0.335 | 0.09910 | 29.58 | 0.141 | 0.529 | 0.3427 | 0.6717 | 90.6 | 16.4 | 6.7 | 2.9 | 35.4 |
| 42003140200 | 0 | -79.92631112 | 40.4478012 | 0.397 | 0.11571 | 29.18 | 0.170 | 0.623 | 0.2232 | 0.5651 | 89.4 | 25.7 | 3.7 | 2.5 | 20 |
| 42003140300 | 1 | -79.91957391 | 40.4414482 | 0.324 | 0.08967 | 27.67 | 0.148 | 0.500 | 0.0469 | 0.7676 | 76 | 7.9 | 2.6 | 1.1 | 21.1 |
| 42003140400 | 1 | -79.91573854 | 40.44933778 | 0.281 | 0.09132 | 32.50 | 0.102 | 0.460 | 0.0166 | 0.6164 | 78.4 | 5.8 | 1.6 | 1.2 | 17 |
| 42003140500 | 1 | -79.90213204 | 40.4516648 | 0.463 | 0.10450 | 22.57 | 0.258 | 0.668 | 0.323 | 0.4114 | 93.4 | 16.4 | 4.8 | 4.3 | 42.1 |
| 42003140600 | 1 | -79.90138099 | 40.44590298 | 0.461 | 0.06656 | 14.44 | 0.331 | 0.591 | 0.1443 | 0.2734 | 79.2 | 13.2 | 2.8 | 2.8 | 14.5 |
| 42003140800 | 1 | -79.91660179 | 40.43327513 | 0.262 | 0.05612 | 21.42 | 0.152 | 0.372 | 0.1931 | 0.8054 | 80.3 | 12 | 4.3 | 2.4 | 19.7 |
| 42003141000 | 0 | -79.89761691 | 40.43289022 | 0.441 | 0.11919 | 27.04 | 0.207 | 0.674 | 0.0551 | 0.0878 | 76.3 | 3.2 | 5 | 1.3 | 4.2 |
| 42003141100 | 0 | -79.90494353 | 40.4206856 | 0.392 | 0.11522 | 29.41 | 0.166 | 0.618 | 0.1725 | 0.5205 | 81.2 | 3.7 | 3.4 | 7.5 | 10.6 |
| 42003141300 | 1 | -79.92821956 | 40.43348404 | 0.280 | 0.07273 | 25.98 | 0.137 | 0.423 | 0.313 | 0.7554 | 88.9 | 23.8 | 1.6 | 5.3 | 33.3 |
| 42003141400 | 1 | -79.91875598 | 40.4213188 | 0.344 | 0.06550 | 19.04 | 0.216 | 0.472 | 0.1189 | 0.7117 | 84 | 10.4 | 3.7 | 2.2 | 24.7 |
| 42003151600 | 1 | -79.9443943 | 40.42500796 | 0.530 | 0.07759 | 14.64 | 0.378 | 0.682 | 0.3196 | 0.5198 | 83.1 | 7.1 | 5.4 | 5.5 | 15.7 |
| 42003151700 | 1 | -79.93312044 | 40.42395144 | 0.368 | 0.09039 | 24.56 | 0.191 | 0.545 | 0.2379 | 0.7341 | 85 | 11.1 | 5.2 | 1.9 | 16.3 |
| 42003160800 | 1 | -79.97108667 | 40.42091908 | 0.515 | 0.09534 | 18.51 | 0.328 | 0.702 | 0.5571 | 0.4952 | 88 | 18.9 | 5.7 | 8.1 | 15.9 |
| 42003160900 | 1 | -79.96427251 | 40.42487946 | 0.328 | 0.07759 | 23.66 | 0.176 | 0.480 | 0.3424 | 0.5308 | 93.1 | 27 | 5.1 | 4.3 | 10.8 |
| 42003170200 | 1 | -79.98491272 | 40.42981368 | 0.434 | 0.09434 | 21.74 | 0.249 | 0.619 | 0.2516 | 0.1615 | 99.1 | 29 | 2.7 | 2.8 | 7.6 |
| 42003170600 | 1 | -79.98460475 | 40.42260103 | 0.586 | 0.10247 | 17.49 | 0.385 | 0.787 | 0.5324 | 0.4367 | 92.5 | 18.8 | 6 | 10.2 | 13.2 |
| 42003180300 | 1 | -79.99377277 | 40.42297321 | 0.645 | 0.09290 | 14.40 | 0.463 | 0.827 | 0.8257 | 0.4292 | 68.9 | 28.2 | 13.7 | 8.1 | 50.7 |
| 42003180700 | 1 | -80.00129287 | 40.42465062 | 0.459 | 0.10232 | 22.29 | 0.258 | 0.660 | 0.4917 | 0.5373 | 84.6 | 22.4 | 3.7 | 7.5 | 25.2 |
| 42003190300 | 1 | -80.01479931 | 40.43274827 | 0.576 | 0.07969 | 13.83 | 0.420 | 0.732 | 0.1214 | 0.1859 | 94 | 11.3 | 2.4 | 3.2 | 8.6 |
| 42003191100 | 1 | -80.0247925 | 40.43542008 | 0.590 | 0.09450 | 16.02 | 0.405 | 0.775 | 0.2307 | 0.4942 | 84.2 | 12.4 | 2.3 | 7.1 | 7.1 |
| 42003191400 | 0 | -80.01109708 | 40.42730329 | 0.455 | 0.11992 | 26.34 | 0.220 | 0.690 | 0.3183 | 0.3789 | 89.4 | 21.7 | 4 | 4.6 | 13 |
| 42003191500 | 0 | -80.01081332 | 40.42052458 | 0.501 | 0.12088 | 24.14 | 0.264 | 0.738 | 0.4504 | 0.3218 | 86.5 | 12.4 | 4.6 | 7 | 19.2 |
| 42003191600 | 1 | -80.023204 | 40.41730668 | 0.509 | 0.06693 | 13.15 | 0.378 | 0.640 | 0.7158 | 0.8516 | 84 | 16.9 | 6.1 | 16.1 | 27.6 |
| 42003191700 | 1 | -80.01243716 | 40.40381032 | 0.403 | 0.08550 | 21.22 | 0.235 | 0.571 | 0.4573 | 0.722 | 80.8 | 7.9 | 7 | 9 | 16.3 |
| 42003191800 | 1 | -80.01633269 | 40.38892686 | 0.609 | 0.05532 | 9.08 | 0.501 | 0.717 | 0.4626 | 0.6492 | 80.3 | 5.9 | 7.5 | 9.5 | 11.6 |
| 42003191900 | 0 | -80.02216624 | 40.39620828 | 0.435 | 0.11885 | 27.32 | 0.202 | 0.668 | 0.5377 | 0.7948 | 81.8 | 13 | 9.1 | 7.1 | 20 |
| 42003192000 | 1 | -80.02704074 | 40.40442637 | 0.395 | 0.10339 | 26.18 | 0.192 | 0.598 | 0.528 | 0.7004 | 80.4 | 14.8 | 6.8 | 5.5 | 20.2 |
| 42003202200 | 1 | -80.06362424 | 40.45405471 | 0.445 | 0.07962 | 17.89 | 0.289 | 0.601 | 0.856 | 0.8001 | 69.7 | 28.5 | 14.2 | 11.3 | 48.6 |
| 42003202300 | 1 | -80.03929629 | 40.41030155 | 0.545 | 0.09055 | 16.62 | 0.368 | 0.722 | 0.1258 | 0.6817 | 90.6 | 5.7 | 2.3 | 6.2 | 20.9 |
| 42003210700 | 1 | -80.02505273 | 40.45550401 | 0.462 | 0.09518 | 20.60 | 0.275 | 0.649 | 0.6391 | 0.4595 | 80.8 | 22.4 | 9.5 | 7 | 73.9 |
| 42003220600 | 1 | -80.01042393 | 40.45606649 | 0.577 | 0.11018 | 19.10 | 0.361 | 0.793 | 0.5186 | 0.4027 | 89.1 | 21.7 | 6.9 | 9.9 | 38.1 |
| 42003240600 | 1 | -79.98264645 | 40.46227214 | 0.522 | 0.11507 | 22.04 | 0.296 | 0.748 | 0.3362 | 0.3483 | 86 | 15.8 | 5.2 | 5.1 | 23.8 |
| 42003241200 | 0 | -79.98921546 | 40.46855195 | 0.561 | 0.11906 | 21.21 | 0.328 | 0.795 | 0.7061 | 0.3555 | 85.1 | 19.3 | 2.5 | 22.8 | 25.2 |
| 42003250300 | 1 | -80.01203255 | 40.4584895 | 0.536 | 0.10139 | 18.92 | 0.337 | 0.735 | 0.6282 | 0.9125 | 73 | 40.8 | 5.6 | 9.6 | 59.4 |
| 42003250700 | 0 | -80.02186174 | 40.45994056 | 0.526 | 0.12055 | 22.91 | 0.290 | 0.762 | 0.9286 | 0.8563 | 74.4 | 42 | 20.1 | 14.1 | 78.2 |
| 42003250900 | 1 | -80.00427591 | 40.46281317 | 0.599 | 0.10700 | 17.86 | 0.389 | 0.809 | 0.7787 | 0.4424 | 81.8 | 22.3 | 19.3 | 6.5 | 60.3 |
| 42003260200 | 1 | -80.01834207 | 40.49139032 | 0.486 | 0.10550 | 21.71 | 0.279 | 0.693 | 0.6304 | 0.3699 | 83 | 19.7 | 7 | 8.7 | 28.6 |
| 42003260700 | 1 | -80.01169566 | 40.4817798 | 0.414 | 0.10941 | 26.43 | 0.200 | 0.628 | 0.4632 | 0.4205 | 81.5 | 4.2 | 8.5 | 11.7 | 46 |
| 42003260900 | 0 | -80.00259646 | 40.47763385 | 0.531 | 0.12042 | 22.68 | 0.295 | 0.767 | 0.9906 | 0.9178 | 59.4 | 75.1 | 35.7 | 22.3 | 97.4 |
| 42003261200 | 0 | -80.0081107 | 40.49223886 | 0.442 | 0.11928 | 26.96 | 0.209 | 0.676 | 0.1446 | 0.2043 | 80.1 | 3.6 | 3.5 | 6.2 | 9.7 |
| 42003261400 | 1 | -80.01206239 | 40.4656585 | 0.499 | 0.10392 | 20.83 | 0.295 | 0.703 | 0.8397 | 0.7223 | 76.7 | 33.9 | 8.5 | 12.2 | 76.1 |
| 42003261500 | 0 | -80.01634937 | 40.47095436 | 0.499 | 0.12088 | 24.23 | 0.262 | 0.736 | 0.7818 | 0.7957 | 79.4 | 31.6 | 11.6 | 7.2 | 55.3 |
| 42003262000 | 0 | -79.99521853 | 40.46717696 | 0.590 | 0.11695 | 19.82 | 0.361 | 0.819 | 0.8504 | 0.4014 | 72.4 | 33.8 | 10 | 12.8 | 37.8 |
| 42003270100 | 1 | -80.04385603 | 40.48321344 | 0.447 | 0.06348 | 14.20 | 0.323 | 0.571 | 0.2817 | 0.3399 | 79.8 | 7.5 | 3.7 | 5.6 | 21.9 |
| 42003270300 | 0 | -80.03675747 | 40.47785096 | 0.559 | 0.11919 | 21.32 | 0.326 | 0.793 | 0.7293 | 0.4002 | 81.1 | 15.9 | 5.9 | 14.6 | 37.5 |
| 42003270400 | 0 | -80.0285115 | 40.46539965 | 0.518 | 0.12072 | 23.29 | 0.282 | 0.755 | 0.7834 | 0.6948 | 87.2 | 19.9 | 9 | 14.1 | 44.4 |
| 42003270800 | 1 | -80.03199705 | 40.48569662 | 0.409 | 0.08444 | 20.65 | 0.243 | 0.575 | 0.5074 | 0.5933 | 84.8 | 16.1 | 11.6 | 4.5 | 22.1 |
| 42003271500 | 1 | -80.02822161 | 40.47302127 | 0.635 | 0.10922 | 17.20 | 0.421 | 0.849 | 0.877 | 0.4155 | 73.8 | 16.3 | 16.7 | 16.8 | 43.4 |
| 42003281400 | 1 | -80.05195822 | 40.44713616 | 0.426 | 0.11476 | 26.94 | 0.201 | 0.651 | 0.7844 | 0.925 | 73.1 | 30.2 | 7 | 11.4 | 52 |
| 42003281500 | 0 | -80.05392954 | 40.44046265 | 0.469 | 0.12043 | 25.67 | 0.233 | 0.705 | 0.3302 | 0.3208 | 78.9 | 6.1 | 4 | 7.7 | 19 |
| 42003290100 | 1 | -79.99317551 | 40.39907485 | 0.514 | 0.10849 | 21.11 | 0.301 | 0.727 | 0.7765 | 0.8394 | 81 | 17 | 13 | 8.2 | 27.5 |
| 42003290200 | 1 | -79.98383155 | 40.39936707 | 0.574 | 0.07443 | 12.97 | 0.428 | 0.720 | 0.8106 | 0.7282 | 78.1 | 18.1 | 12.4 | 12.1 | 20.3 |
| 42003290400 | 1 | -79.98248302 | 40.38765826 | 0.655 | 0.08438 | 12.88 | 0.490 | 0.820 | 0.8147 | 0.5548 | 77.6 | 22.7 | 14.5 | 13.6 | 28.4 |
| 42003300100 | 1 | -79.99300725 | 40.41355501 | 0.535 | 0.09290 | 17.36 | 0.353 | 0.717 | 0.9377 | 0.8326 | 66.3 | 39.8 | 16.6 | 16.9 | 70.3 |
| 42003310200 | 1 | -79.91411311 | 40.37140335 | 0.493 | 0.06760 | 13.71 | 0.361 | 0.625 | 0.57 | 0.1537 | 79.1 | 6.3 | 11 | 7.7 | 7.1 |
| 42003310300 | 0 | -79.9225466 | 40.38792622 | 0.456 | 0.11994 | 26.32 | 0.221 | 0.691 | 0.3853 | 0.4702 | 79.1 | 2.9 | 6.6 | 11.2 | 11.3 |
| 42003320400 | 0 | -79.99204611 | 40.38470088 | 0.546 | 0.11987 | 21.97 | 0.311 | 0.781 | 0.6729 | 0.3936 | 79.7 | 20.3 | 8.5 | 6 | 8.4 |
| 42003320600 | 0 | -80.00506448 | 40.39466782 | 0.442 | 0.11928 | 26.96 | 0.209 | 0.676 | 0.4879 | 0.6854 | 78.6 | 14.4 | 9.8 | 4.1 | 13.1 |
| 42003320700 | 0 | -80.0002366 | 40.38245862 | 0.560 | 0.11914 | 21.27 | 0.327 | 0.794 | 0.7421 | 0.4136 | 83.6 | 16.2 | 8.1 | 13.7 | 9.9 |
| 42003401100 | 1 | -79.71009649 | 40.6461345 | 0.424 | 0.06458 | 15.23 | 0.297 | 0.551 | 0.4385 | 0.4724 | 80.3 | 7 | 7 | 6.7 | 5 |
| 42003401200 | 0 | -79.72540602 | 40.61501866 | 0.629 | 0.11279 | 17.92 | 0.408 | 0.850 | 0.8041 | 0.1172 | 79.1 | 32.3 | 11.7 | 7.2 | 5.5 |
| 42003401300 | 1 | -79.73888382 | 40.62152569 | 0.432 | 0.11384 | 26.35 | 0.209 | 0.655 | 0.2814 | 0.1809 | 82.4 | 3.1 | 7.3 | 3.4 | 8.4 |
| 42003402000 | 1 | -79.74106169 | 40.60794104 | 0.519 | 0.08408 | 16.20 | 0.354 | 0.684 | 0.5383 | 0.3946 | 80.5 | 13.2 | 7.7 | 8.2 | 8.5 |
| 42003403500 | 1 | -79.76010137 | 40.6041269 | 0.525 | 0.08978 | 17.10 | 0.349 | 0.701 | 0.6873 | 0.2493 | 77.3 | 18.4 | 6.9 | 8 | 12.9 |
| 42003404000 | 1 | -79.78004966 | 40.58812263 | 0.625 | 0.10188 | 16.30 | 0.425 | 0.825 | 0.4516 | 0.2337 | 81.5 | 9.6 | 7.8 | 6.7 | 3.6 |
| 42003405000 | 1 | -79.79642507 | 40.60650201 | 0.421 | 0.10100 | 23.99 | 0.223 | 0.619 | 0.2701 | 0.2021 | 82.7 | 8.9 | 4.6 | 6.2 | 1 |
| 42003406000 | 0 | -79.76736559 | 40.649976 | 0.473 | 0.12054 | 25.48 | 0.237 | 0.709 | 0.3236 | 0.2908 | 84.6 | 4.9 | 6.4 | 7.2 | 3 |
| 42003407001 | 1 | -79.86850993 | 40.64759213 | 0.511 | 0.08843 | 17.31 | 0.338 | 0.684 | 0.3293 | 0.044 | 80 | 1.8 | 6.4 | 9.8 | 2.5 |
| 42003407002 | 1 | -79.87073667 | 40.61174926 | 0.529 | 0.07810 | 14.76 | 0.376 | 0.682 | 0.1734 | 0.0194 | 80.2 | 6.1 | 3.5 | 5.4 | 1.3 |
| 42003408001 | 1 | -79.95927214 | 40.65280441 | 0.432 | 0.07707 | 17.84 | 0.281 | 0.583 | 0.2059 | 0.1028 | 76.9 | 7.7 | 4.8 | 6 | 4.9 |
| 42003408002 | 1 | -79.95498239 | 40.62412772 | 0.450 | 0.05983 | 13.30 | 0.333 | 0.567 | 0.0269 | 0.3549 | 74.1 | 4.1 | 2.5 | 1.2 | 8.8 |
| 42003409000 | 1 | -80.03146823 | 40.64370961 | 0.248 | 0.04438 | 17.90 | 0.161 | 0.335 | 0.0444 | 0.2787 | 71 | 2.8 | 5.2 | 0.7 | 6.9 |
| 42003410000 | 1 | -80.08089745 | 40.63693568 | 0.351 | 0.10198 | 29.05 | 0.151 | 0.551 | 0.0066 | 0.0834 | 78 | 2.2 | 2.4 | 2.3 | 4.1 |
| 42003411000 | 1 | -80.11003302 | 40.64534399 | 0.428 | 0.07120 | 16.64 | 0.288 | 0.568 | 0.071 | 0.582 | 70.9 | 2.2 | 5.7 | 2.3 | 12.3 |
| 42003412001 | 1 | -80.1071271 | 40.60099273 | 0.295 | 0.04438 | 15.05 | 0.208 | 0.382 | 0.0592 | 0.7229 | 72.3 | 1.7 | 5.7 | 1.2 | 18.1 |
| 42003412002 | 1 | -80.08683651 | 40.57114307 | 0.202 | 0.05376 | 26.61 | 0.097 | 0.307 | 0.0022 | 0.5098 | 71.4 | 1.1 | 2.7 | 0.7 | 10.7 |
| 42003413100 | 1 | -80.02993319 | 40.60184131 | 0.266 | 0.08922 | 33.54 | 0.091 | 0.441 | 0.0764 | 0.8179 | 74.8 | 7.2 | 3.9 | 1.3 | 26.4 |
| 42003413201 | 0 | -80.04060102 | 40.57602157 | 0.386 | 0.11465 | 29.67 | 0.162 | 0.611 | 0.0532 | 0.383 | 82 | 4.3 | 2.2 | 2.8 | 5.6 |
| 42003413202 | 0 | -80.05802563 | 40.58072319 | 0.353 | 0.11037 | 31.31 | 0.136 | 0.569 | 0.0341 | 0.5498 | 77.6 | 4.3 | 2.7 | 2.5 | 4.3 |
| 42003413300 | 1 | -80.04312618 | 40.56094349 | 0.395 | 0.04950 | 12.53 | 0.298 | 0.492 | 0.1246 | 0.4283 | 79.7 | 4.1 | 4 | 5.3 | 10.2 |
| 42003413400 | 0 | -80.00534842 | 40.56096143 | 0.388 | 0.11485 | 29.58 | 0.163 | 0.613 | 0.0394 | 0.3536 | 78.2 | 2.6 | 2.3 | 4.6 | 8.5 |
| 42003413500 | 1 | -80.00783717 | 40.58058696 | 0.313 | 0.09602 | 30.68 | 0.125 | 0.501 | 0.1894 | 0.6182 | 84.8 | 6 | 5 | 3.6 | 13.4 |
| 42003414101 | 1 | -79.93343683 | 40.5976543 | 0.397 | 0.08444 | 21.27 | 0.231 | 0.563 | 0.0044 | 0.0247 | 76.7 | 2 | 0.7 | 1.5 | 1.6 |
| 42003414102 | 1 | -79.9700175 | 40.59192691 | 0.442 | 0.05975 | 13.52 | 0.325 | 0.559 | 0.0382 | 0.542 | 77 | 3.6 | 1.6 | 4.6 | 9.6 |
| 42003414200 | 1 | -79.94552303 | 40.56578101 | 0.362 | 0.06986 | 19.30 | 0.225 | 0.499 | 0.1947 | 0.1406 | 75.3 | 6.9 | 6.5 | 2.6 | 6.4 |
| 42003415001 | 1 | -79.86283217 | 40.5808394 | 0.326 | 0.08093 | 24.83 | 0.167 | 0.485 | 0.1117 | 0.5392 | 75.6 | 6.6 | 3 | 5.7 | 10.8 |
| 42003415002 | 0 | -79.90069884 | 40.55356328 | 0.435 | 0.11887 | 27.30 | 0.202 | 0.668 | 0.1164 | 0.2027 | 79.8 | 3.1 | 6.1 | 4.3 | 9.5 |
| 42003416000 | 1 | -79.78720093 | 40.55435617 | 0.492 | 0.10359 | 21.05 | 0.289 | 0.695 | 0.369 | 0.119 | 88 | 12.7 | 4.4 | 7.1 | 0.9 |
| 42003417100 | 1 | -79.7844336 | 40.54414717 | 0.530 | 0.09747 | 18.39 | 0.339 | 0.721 | 0.2272 | 0.0706 | 88 | 8.3 | 3.3 | 4.1 | 3.6 |
| 42003417200 | 1 | -79.78029022 | 40.53878229 | 0.527 | 0.10995 | 20.86 | 0.311 | 0.743 | 0.6864 | 0.0034 | 80.6 | 18 | 14.1 | 10 | 0.1 |
| 42003418000 | 0 | -79.80124345 | 40.54277389 | 0.534 | 0.12033 | 22.54 | 0.298 | 0.770 | 0.3562 | 0.0131 | 84.3 | 8.8 | 8.6 | 3.7 | 1 |
| 42003419000 | 1 | -79.83870466 | 40.54704342 | 0.558 | 0.07396 | 13.25 | 0.413 | 0.703 | 0.3158 | 0.0884 | 86.2 | 8.6 | 5.4 | 8.3 | 4.3 |
| 42003420000 | 0 | -79.85994119 | 40.4921867 | 0.423 | 0.11800 | 27.91 | 0.191 | 0.654 | 0.5027 | 0.8129 | 79 | 12.6 | 3.4 | 10 | 20 |
| 42003421100 | 1 | -79.86689603 | 40.50453915 | 0.364 | 0.05745 | 15.78 | 0.251 | 0.477 | 0.1058 | 0.4908 | 77.2 | 4.6 | 4.9 | 3.9 | 18.1 |
| 42003421200 | 1 | -79.92368025 | 40.51508138 | 0.351 | 0.10602 | 30.20 | 0.143 | 0.559 | 0.0397 | 0.3486 | 79.8 | 2.5 | 3.7 | 3.8 | 8 |
| 42003422000 | 1 | -79.88979183 | 40.52471678 | 0.307 | 0.05301 | 17.27 | 0.203 | 0.411 | 0.1834 | 0.2527 | 69.8 | 4.7 | 9.4 | 1.2 | 13.1 |
| 42003423000 | 1 | -79.90374691 | 40.49287512 | 0.350 | 0.09742 | 27.83 | 0.159 | 0.541 | 0.1402 | 0.6826 | 78 | 4.7 | 5.7 | 4.2 | 9.3 |
| 42003424000 | 1 | -79.92272743 | 40.49341651 | 0.540 | 0.09752 | 18.06 | 0.349 | 0.731 | 0.8191 | 0.8169 | 82.2 | 25.5 | 12.8 | 11 | 20.9 |
| 42003425000 | 1 | -79.94754168 | 40.49760261 | 0.531 | 0.08385 | 15.79 | 0.367 | 0.695 | 0.4335 | 0.4217 | 82 | 10.5 | 4.9 | 7 | 5.5 |
| 42003426300 | 1 | -79.97458888 | 40.54395573 | 0.496 | 0.07369 | 14.86 | 0.352 | 0.640 | 0.1061 | 0.2174 | 80 | 3 | 4.5 | 4 | 3.1 |
| 42003426400 | 1 | -79.95989311 | 40.52192211 | 0.424 | 0.06950 | 16.39 | 0.288 | 0.560 | 0.318 | 0.4102 | 87.9 | 8.8 | 7.4 | 3.5 | 4.3 |
| 42003426700 | 0 | -79.94334661 | 40.51636299 | 0.532 | 0.12040 | 22.64 | 0.296 | 0.768 | 0.4185 | 0.1115 | 86.8 | 6.4 | 6.3 | 12.1 | 5.3 |
| 42003426800 | 1 | -79.9500496 | 40.54204542 | 0.398 | 0.09581 | 24.07 | 0.210 | 0.586 | 0.1017 | 0.209 | 80.3 | 3.2 | 3.7 | 5.3 | 1.9 |
| 42003427000 | 1 | -79.97361029 | 40.48168043 | 0.583 | 0.06804 | 11.67 | 0.450 | 0.716 | 0.6006 | 0.413 | 82.4 | 23.7 | 4.3 | 9.3 | 7.5 |
| 42003427100 | 1 | -79.97690582 | 40.51666775 | 0.561 | 0.09445 | 16.84 | 0.376 | 0.746 | 0.19 | 0.1996 | 80.7 | 5.6 | 4.9 | 5.1 | 2.1 |
| 42003427200 | 1 | -79.96521894 | 40.49454956 | 0.399 | 0.07842 | 19.65 | 0.245 | 0.553 | 0.4657 | 0.3449 | 81.3 | 11.4 | 9.8 | 5.8 | 5.4 |
| 42003428100 | 0 | -79.98085354 | 40.47392339 | 0.549 | 0.11972 | 21.80 | 0.314 | 0.784 | 0.4131 | 0.0116 | 84.8 | 5.4 | 7.4 | 6.3 | 0.9 |
| 42003428200 | 0 | -79.99047285 | 40.48742626 | 0.480 | 0.12069 | 25.16 | 0.243 | 0.716 | 0.21 | 0.0965 | 83.3 | 4.5 | 3.9 | 8 | 4.6 |
| 42003429100 | 0 | -80.00772252 | 40.50625324 | 0.368 | 0.11249 | 30.55 | 0.148 | 0.589 | 0.2163 | 0.7145 | 80.7 | 7.5 | 5.2 | 5.5 | 13.4 |
| 42003429201 | 0 | -80.00366702 | 40.54471886 | 0.340 | 0.10848 | 31.92 | 0.127 | 0.552 | 0.1277 | 0.7557 | 82.6 | 4.1 | 1.6 | 7.9 | 16.1 |
| 42003429202 | 1 | -79.9952454 | 40.52435647 | 0.411 | 0.11283 | 27.45 | 0.190 | 0.632 | 0.0573 | 0.6192 | 79.5 | 3.8 | 3.8 | 1.7 | 11.6 |
| 42003429300 | 1 | -80.05064555 | 40.53682999 | 0.383 | 0.05514 | 14.40 | 0.275 | 0.491 | 0.1362 | 0.0912 | 85.1 | 1.5 | 5.6 | 5.1 | 4.4 |
| 42003429400 | 0 | -80.02756861 | 40.53278072 | 0.447 | 0.11952 | 26.74 | 0.213 | 0.681 | 0.1484 | 0.1853 | 78.9 | 6.4 | 4.3 | 3.7 | 8.5 |
| 42003429500 | 1 | -80.01809365 | 40.53692421 | 0.430 | 0.05701 | 13.26 | 0.318 | 0.542 | 0.2448 | 0.2896 | 90.5 | 5.2 | 3.6 | 11.6 | 16.1 |
| 42003429600 | 1 | -80.04804949 | 40.50968672 | 0.370 | 0.10100 | 27.30 | 0.172 | 0.568 | 0.1765 | 0.3833 | 81.6 | 8 | 3.3 | 4.4 | 7.3 |
| 42003429700 | 0 | -80.03393469 | 40.49924639 | 0.477 | 0.12063 | 25.29 | 0.241 | 0.713 | 0.1881 | 0.0803 | 80.6 | 7.3 | 2.4 | 3.7 | 3.9 |
| 42003430100 | 0 | -80.02474937 | 40.5145876 | 0.478 | 0.12065 | 25.23 | 0.242 | 0.715 | 0.2573 | 0.1712 | 80.9 | 7.7 | 4 | 6.7 | 7.9 |
| 42003430200 | 1 | -80.03744658 | 40.51988121 | 0.529 | 0.09518 | 17.99 | 0.342 | 0.716 | 0.2635 | 0.2774 | 81.8 | 7.4 | 5.1 | 6.9 | 2.8 |
| 42003431100 | 1 | -80.05064346 | 40.49014681 | 0.418 | 0.11229 | 26.86 | 0.198 | 0.638 | 0.452 | 0.5067 | 84 | 16.9 | 6.8 | 4.1 | 17.7 |
| 42003431400 | 0 | -80.05523374 | 40.50295614 | 0.504 | 0.12088 | 23.96 | 0.267 | 0.741 | 0.4197 | 0.259 | 84.2 | 13 | 5.5 | 5.5 | 13.5 |
| 42003431500 | 1 | -80.05796311 | 40.49306104 | 0.428 | 0.08683 | 20.29 | 0.258 | 0.598 | 0.5117 | 0.2234 | 90 | 15.3 | 4.5 | 9.1 | 11 |
| 42003432300 | 1 | -80.06785403 | 40.50458598 | 0.456 | 0.10909 | 23.92 | 0.242 | 0.670 | 0.4341 | 0.5895 | 84.7 | 11.4 | 5.4 | 7.3 | 18.4 |
| 42003432400 | 1 | -80.06820809 | 40.49767605 | 0.448 | 0.09471 | 21.14 | 0.262 | 0.634 | 0.4178 | 0.2693 | 81.5 | 8.9 | 7.3 | 3.9 | 14 |
| 42003434000 | 1 | -80.0814818 | 40.50582579 | 0.327 | 0.09823 | 30.04 | 0.134 | 0.520 | 0.0175 | 0.2746 | 76.5 | 2.9 | 3 | 2.2 | 5.2 |
| 42003435000 | 0 | -80.09656089 | 40.5112233 | 0.479 | 0.12068 | 25.18 | 0.243 | 0.716 | 0.2513 | 0.1571 | 80.9 | 5 | 5.1 | 6.5 | 7.4 |
| 42003437000 | 1 | -80.09258346 | 40.54248518 | 0.303 | 0.08479 | 27.98 | 0.137 | 0.469 | 0.0798 | 0.2777 | 75.6 | 4.3 | 3.8 | 5 | 15.2 |
| 42003439000 | 0 | -80.17379719 | 40.58977014 | 0.431 | 0.11858 | 27.52 | 0.199 | 0.663 | 0.067 | 0.1575 | 74.7 | 7.3 | 2.8 | 3.2 | 7.5 |
| 42003445500 | 1 | -80.17932779 | 40.53992019 | 0.294 | 0.07071 | 24.05 | 0.155 | 0.433 | 0.0698 | 0.5061 | 77 | 7 | 3 | 3.3 | 11.8 |
| 42003446000 | 0 | -80.19221239 | 40.55461045 | 0.421 | 0.11788 | 27.99 | 0.190 | 0.652 | 0.0088 | 0.129 | 70.4 | 2.4 | 3.7 | 0.6 | 5.9 |
| 42003447000 | 0 | -80.20377777 | 40.57401615 | 0.362 | 0.11166 | 30.86 | 0.143 | 0.581 | 0.0297 | 0.4898 | 73.5 | 3 | 1.5 | 3.5 | 5.9 |
| 42003448000 | 1 | -80.21421455 | 40.56563374 | 0.363 | 0.09290 | 25.59 | 0.181 | 0.545 | 0.466 | 0.702 | 76.1 | 19.3 | 2.8 | 9.4 | 23 |
| 42003449000 | 1 | -80.22856268 | 40.55626702 | 0.461 | 0.08746 | 18.97 | 0.290 | 0.632 | 0.2028 | 0.3833 | 79.5 | 6 | 3.4 | 6.1 | 6.3 |
| 42003450700 | 0 | -80.1587583 | 40.5110125 | 0.433 | 0.11871 | 27.42 | 0.200 | 0.666 | 0.3762 | 0.5798 | 84.5 | 10.2 | 5.1 | 4.8 | 15.3 |
| 42003450800 | 0 | -80.16758556 | 40.51969564 | 0.510 | 0.12084 | 23.69 | 0.273 | 0.747 | 0.8729 | 0.8641 | 79.1 | 25.2 | 14.6 | 16.3 | 36.1 |
| 42003451101 | 1 | -80.21851364 | 40.53747523 | 0.343 | 0.09849 | 28.71 | 0.150 | 0.536 | 0.0808 | 0.1415 | 78 | 4.6 | 4.9 | 1.8 | 6.5 |
| 42003451102 | 1 | -80.19895554 | 40.52274983 | 0.348 | 0.09879 | 28.39 | 0.154 | 0.542 | 0.5255 | 0.7251 | 86.7 | 8.7 | 7.1 | 8.9 | 16.9 |
| 42003451104 | 0 | -80.25377623 | 40.53757899 | 0.388 | 0.11485 | 29.58 | 0.163 | 0.613 | 0.1346 | 0.487 | 75.2 | 3.5 | 6.5 | 2.2 | 7.3 |
| 42003451105 | 1 | -80.23526669 | 40.51432665 | 0.378 | 0.10040 | 26.56 | 0.181 | 0.575 | 0.6357 | 0.856 | 79.9 | 16.9 | 9.1 | 9.8 | 24.1 |
| 42003451300 | 1 | -80.18601826 | 40.4886727 | 0.447 | 0.04347 | 9.73 | 0.362 | 0.532 | 0.0147 | 0.5095 | 77.4 | 0.4 | 3.1 | 2.9 | 10.1 |
| 42003452000 | 1 | -80.28114511 | 40.47824422 | 0.447 | 0.08591 | 19.22 | 0.279 | 0.615 | 0.1412 | 0.3008 | 75.1 | 5.5 | 3.9 | 4.6 | 4.7 |
| 42003453003 | 0 | -80.19186623 | 40.4258034 | 0.423 | 0.11800 | 27.91 | 0.192 | 0.654 | 0.1865 | 0.3693 | 75.5 | 3.6 | 4.8 | 4.8 | 4.3 |
| 42003453004 | 1 | -80.2028598 | 40.44870491 | 0.559 | 0.07369 | 13.18 | 0.415 | 0.703 | 0.1621 | 0.478 | 81.4 | 10.7 | 2.9 | 4.3 | 8.4 |
| 42003455000 | 0 | -80.18715134 | 40.40016542 | 0.466 | 0.12033 | 25.81 | 0.230 | 0.702 | 0.2297 | 0.1959 | 82.3 | 6.6 | 4.4 | 5.7 | 3.7 |
| 42003456001 | 0 | -80.15178662 | 40.36903314 | 0.430 | 0.11852 | 27.56 | 0.198 | 0.662 | 0.1559 | 0.2868 | 74.9 | 8.3 | 3.2 | 2.6 | 4.7 |
| 42003456003 | 1 | -80.17439634 | 40.35509503 | 0.326 | 0.05805 | 17.81 | 0.212 | 0.440 | 0.098 | 0.3808 | 70.5 | 3 | 4.5 | 6.1 | 11.4 |
| 42003456004 | 1 | -80.12264886 | 40.3284839 | 0.429 | 0.07772 | 18.12 | 0.277 | 0.581 | 0.2175 | 0.2887 | 80.2 | 4.6 | 5.7 | 6.3 | 3 |
| 42003457100 | 0 | -80.11400537 | 40.36124121 | 0.556 | 0.11939 | 21.48 | 0.322 | 0.790 | 0.6338 | 0.2852 | 83.6 | 14.8 | 7.5 | 10.1 | 6.8 |
| 42003457200 | 1 | -80.10216119 | 40.35602572 | 0.502 | 0.08927 | 17.78 | 0.327 | 0.677 | 0.3261 | 0.2862 | 83.8 | 8.3 | 6 | 5.8 | 5.1 |
| 42003458000 | 1 | -80.13205735 | 40.39910617 | 0.567 | 0.04950 | 8.73 | 0.470 | 0.664 | 0.1421 | 0.2715 | 83.7 | 5 | 6.2 | 2.4 | 5.4 |
| 42003459101 | 0 | -80.13544699 | 40.49699148 | 0.560 | 0.11913 | 21.27 | 0.327 | 0.794 | 0.6664 | 0.3065 | 74.7 | 15.6 | 10.1 | 10.7 | 17.4 |
| 42003459102 | 1 | -80.13626638 | 40.47094597 | 0.439 | 0.06050 | 13.78 | 0.320 | 0.558 | 0.1512 | 0.4267 | 84.7 | 4.8 | 1.6 | 10.2 | 4.8 |
| 42003459201 | 1 | -80.13142048 | 40.44156779 | 0.517 | 0.09990 | 19.32 | 0.321 | 0.713 | 0.0773 | 0.7498 | 86 | 5.9 | 4 | 2.6 | 13.5 |
| 42003459202 | 0 | -80.1010937 | 40.42824515 | 0.375 | 0.11333 | 30.22 | 0.153 | 0.597 | 0.0479 | 0.4402 | 91.7 | 6.6 | 1 | 1.7 | 9.1 |
| 42003460001 | 1 | -80.10520103 | 40.48838095 | 0.581 | 0.06565 | 11.30 | 0.452 | 0.710 | 0.1274 | 0.2871 | 82.5 | 6.6 | 2.8 | 4.4 | 3.4 |
| 42003460002 | 0 | -80.10070852 | 40.46631221 | 0.413 | 0.11720 | 28.40 | 0.183 | 0.642 | 0.1365 | 0.3546 | 80.4 | 3.7 | 4.7 | 4.3 | 8.1 |
| 42003461000 | 1 | -80.11150447 | 40.50634563 | 0.471 | 0.09864 | 20.94 | 0.278 | 0.664 | 0.4911 | 0.4645 | 86.6 | 12.9 | 5.2 | 9.9 | 8.9 |
| 42003462100 | 1 | -80.07305237 | 40.48489142 | 0.600 | 0.10109 | 16.85 | 0.402 | 0.798 | 0.9158 | 0.3733 | 74.9 | 37.3 | 20 | 15 | 29.6 |
| 42003462600 | 1 | -80.07496183 | 40.47404634 | 0.588 | 0.08485 | 14.43 | 0.422 | 0.754 | 0.7515 | 0.5848 | 78.1 | 21 | 8.5 | 9.7 | 27.8 |
| 42003463900 | 0 | -80.07279119 | 40.46626781 | 0.597 | 0.11633 | 19.48 | 0.369 | 0.825 | 0.852 | 0.3655 | 83 | 22.1 | 10 | 16.6 | 26.8 |
| 42003464300 | 1 | -80.06850546 | 40.44483184 | 0.519 | 0.08081 | 15.57 | 0.361 | 0.677 | 0.334 | 0.2362 | 82.1 | 11.3 | 5.5 | 5.3 | 11.8 |
| 42003464400 | 0 | -80.05780918 | 40.47008454 | 0.586 | 0.11730 | 20.01 | 0.356 | 0.816 | 0.8372 | 0.4052 | 74.2 | 28.1 | 8.3 | 16.6 | 39.3 |
| 42003465600 | 0 | -80.06841302 | 40.43853375 | 0.472 | 0.12050 | 25.54 | 0.236 | 0.708 | 0.3296 | 0.3058 | 85.8 | 8.3 | 5.2 | 4.3 | 17.3 |
| 42003465800 | 1 | -80.07248798 | 40.43065028 | 0.464 | 0.08289 | 17.86 | 0.302 | 0.626 | 0.0682 | 0.2081 | 82 | 2.9 | 2.9 | 3.9 | 9.8 |
| 42003468700 | 1 | -80.0887816 | 40.4137854 | 0.473 | 0.11576 | 24.47 | 0.246 | 0.700 | 0.4679 | 0.1993 | 81.9 | 16.1 | 6.4 | 4.6 | 9.4 |
| 42003468800 | 0 | -80.09454159 | 40.40649056 | 0.519 | 0.12071 | 23.25 | 0.283 | 0.756 | 0.5102 | 0.3077 | 82.9 | 19.8 | 6.3 | 6.9 | 17.5 |
| 42003468900 | 1 | -80.07823221 | 40.40407539 | 0.391 | 0.07050 | 18.03 | 0.253 | 0.529 | 0.5393 | 0.6901 | 75 | 24.8 | 8 | 5.6 | 23 |
| 42003469000 | 1 | -80.05431557 | 40.41705708 | 0.316 | 0.06229 | 19.71 | 0.194 | 0.438 | 0.18 | 0.4536 | 82.4 | 8.2 | 4.7 | 3.7 | 7.2 |
| 42003470300 | 1 | -80.08227166 | 40.38156886 | 0.296 | 0.08695 | 29.37 | 0.126 | 0.466 | 0.2266 | 0.7354 | 84.2 | 8.1 | 3.8 | 7.5 | 13 |
| 42003470400 | 0 | -80.08746886 | 40.36855267 | 0.456 | 0.11996 | 26.30 | 0.221 | 0.691 | 0.1743 | 0.1721 | 82.4 | 1.5 | 5.8 | 4.7 | 7.9 |
| 42003470501 | 1 | -80.05944461 | 40.39761623 | 0.254 | 0.06116 | 24.08 | 0.134 | 0.374 | 0.1609 | 0.855 | 84.3 | 8.7 | 4.5 | 3.4 | 40.5 |
| 42003470502 | 1 | -80.06978616 | 40.40268026 | 0.410 | 0.07817 | 19.06 | 0.257 | 0.563 | 0.0876 | 0.1681 | 85.1 | 2 | 4.2 | 5.8 | 7.8 |
| 42003470600 | 1 | -80.0868307 | 40.39532434 | 0.503 | 0.07887 | 15.68 | 0.348 | 0.658 | 0.5387 | 0.4061 | 79.2 | 12.8 | 7.2 | 4.3 | 8.3 |
| 42003471000 | 0 | -80.09220667 | 40.39146733 | 0.427 | 0.11832 | 27.70 | 0.195 | 0.659 | 0.3343 | 0.5523 | 84.6 | 7.1 | 2.6 | 9 | 5.7 |
| 42003472100 | 0 | -80.03592622 | 40.39915494 | 0.390 | 0.11506 | 29.49 | 0.165 | 0.616 | 0.1383 | 0.4814 | 85 | 5.9 | 5.2 | 2.3 | 8 |
| 42003472200 | 0 | -80.04144761 | 40.39456201 | 0.453 | 0.11981 | 26.46 | 0.218 | 0.688 | 0.1174 | 0.1106 | 86.7 | 6.7 | 2.8 | 3.1 | 5.2 |
| 42003472300 | 1 | -80.03300411 | 40.39254855 | 0.398 | 0.10817 | 27.18 | 0.186 | 0.610 | 0.4936 | 0.5633 | 87.3 | 9.7 | 7.3 | 6.9 | 9.3 |
| 42003472400 | 0 | -80.03806855 | 40.38785366 | 0.464 | 0.12026 | 25.92 | 0.228 | 0.700 | 0.1293 | 0.0669 | 80.7 | 1.6 | 5.6 | 2.6 | 3.4 |
| 42003473100 | 1 | -80.05048579 | 40.3927592 | 0.307 | 0.08701 | 28.34 | 0.136 | 0.478 | 0.0782 | 0.6507 | 74.9 | 5.7 | 4.3 | 2.1 | 9 |
| 42003473200 | 1 | -80.04832355 | 40.38147073 | 0.352 | 0.08916 | 25.33 | 0.177 | 0.527 | 0.0848 | 0.6601 | 79 | 8.5 | 2 | 3 | 13.4 |
| 42003473300 | 1 | -80.03029319 | 40.38004088 | 0.444 | 0.08361 | 18.83 | 0.280 | 0.608 | 0.0423 | 0.2231 | 74.6 | 4.9 | 2.6 | 2.9 | 4.8 |
| 42003473401 | 1 | -80.04262074 | 40.37182342 | 0.279 | 0.04930 | 17.67 | 0.182 | 0.376 | 0.0989 | 0.6136 | 74.1 | 8.4 | 3.3 | 3.6 | 8.5 |
| 42003473402 | 0 | -80.04174069 | 40.35916673 | 0.375 | 0.11338 | 30.20 | 0.153 | 0.598 | 0.0038 | 0.3761 | 78 | 2.8 | 2.1 | 0.8 | 8.4 |
| 42003473500 | 0 | -80.05456558 | 40.36574283 | 0.385 | 0.11447 | 29.75 | 0.160 | 0.609 | 0.1678 | 0.553 | 72.7 | 4.8 | 7.7 | 1.3 | 5.9 |
| 42003473601 | 1 | -80.06181431 | 40.38439338 | 0.335 | 0.07759 | 23.16 | 0.183 | 0.487 | 0.0072 | 0.4977 | 76 | 3.7 | 2 | 1.4 | 10.2 |
| 42003473602 | 1 | -80.06920118 | 40.37138972 | 0.393 | 0.05310 | 13.51 | 0.289 | 0.497 | 0.0601 | 0.4467 | 80.1 | 7.1 | 3.9 | 0.6 | 13.1 |
| 42003474101 | 0 | -80.07541696 | 40.35622169 | 0.339 | 0.10842 | 31.94 | 0.127 | 0.552 | 0.0232 | 0.6114 | 73 | 4.9 | 2.5 | 1.9 | 13.6 |
| 42003474102 | 0 | -80.06734801 | 40.34029569 | 0.422 | 0.11793 | 27.96 | 0.191 | 0.653 | 0.0191 | 0.14 | 70.5 | 2.8 | 3.7 | 1.7 | 6.4 |
| 42003474201 | 0 | -80.09620534 | 40.34463261 | 0.390 | 0.11500 | 29.51 | 0.164 | 0.615 | 0.0376 | 0.3433 | 74.6 | 1.5 | 5.2 | 0 | 7.6 |
| 42003474202 | 1 | -80.09530651 | 40.32533876 | 0.349 | 0.05187 | 14.86 | 0.247 | 0.451 | 0.0185 | 0.433 | 74.4 | 1.8 | 4.3 | 0.8 | 8.6 |
| 42003474203 | 1 | -80.07910757 | 40.31377614 | 0.315 | 0.10104 | 32.08 | 0.117 | 0.513 | 0.0448 | 0.5961 | 73.4 | 3.8 | 4 | 1.9 | 15.2 |
| 42003475101 | 1 | -80.04045936 | 40.34696992 | 0.358 | 0.09597 | 26.81 | 0.170 | 0.546 | 0.1781 | 0.5611 | 85.2 | 8.4 | 1.9 | 7.5 | 10 |
| 42003475102 | 0 | -80.03613776 | 40.33703686 | 0.465 | 0.12029 | 25.87 | 0.229 | 0.701 | 0.1615 | 0.1068 | 81 | 5.1 | 4.3 | 5.7 | 5.1 |
| 42003475200 | 1 | -80.05600683 | 40.32402355 | 0.402 | 0.10904 | 27.12 | 0.188 | 0.616 | 0.2222 | 0.1568 | 78.8 | 7.7 | 6 | 2.5 | 2.6 |
| 42003475301 | 1 | -80.03403613 | 40.32421627 | 0.299 | 0.07382 | 24.69 | 0.154 | 0.444 | 0.1825 | 0.5589 | 81.4 | 3.8 | 3.3 | 7.1 | 10.6 |
| 42003475303 | 1 | -80.04676119 | 40.30812569 | 0.422 | 0.05030 | 11.92 | 0.323 | 0.521 | 0.1474 | 0.3933 | 79 | 4.4 | 5.8 | 2.2 | 2.7 |
| 42003475304 | 1 | -80.04835684 | 40.29922854 | 0.343 | 0.07253 | 21.14 | 0.201 | 0.485 | 0.0898 | 0.1928 | 81.5 | 2.9 | 4.7 | 4.3 | 8.9 |
| 42003475401 | 1 | -80.01475552 | 40.34034655 | 0.361 | 0.10714 | 29.68 | 0.151 | 0.571 | 0.0801 | 0.3421 | 79 | 4.2 | 2.4 | 4 | 1.5 |
| 42003475402 | 0 | -80.02271393 | 40.31325111 | 0.445 | 0.11945 | 26.82 | 0.211 | 0.680 | 0.0413 | 0.0434 | 77 | 4.6 | 3.3 | 0.4 | 2.5 |
| 42003476100 | 1 | -80.02295237 | 40.36760745 | 0.513 | 0.06301 | 12.28 | 0.390 | 0.636 | 0.3922 | 0.7644 | 85.2 | 15.1 | 4.5 | 6.1 | 13.4 |
| 42003476200 | 1 | -80.01451525 | 40.36477062 | 0.452 | 0.10421 | 23.06 | 0.248 | 0.656 | 0.3552 | 0.5345 | 82.5 | 14.5 | 5.2 | 5.2 | 6.6 |
| 42003477100 | 1 | -80.00501419 | 40.35849128 | 0.469 | 0.09701 | 20.68 | 0.279 | 0.659 | 0.1937 | 0.2299 | 80.7 | 9.3 | 4.2 | 3.9 | 1 |
| 42003477200 | 1 | -79.99332284 | 40.36582173 | 0.447 | 0.09664 | 21.62 | 0.258 | 0.636 | 0.2106 | 0.1281 | 83.6 | 3.4 | 6.9 | 3.8 | 0.5 |
| 42003477300 | 1 | -79.97764981 | 40.35666614 | 0.454 | 0.04960 | 10.92 | 0.357 | 0.551 | 0.5728 | 0.8847 | 79 | 24.8 | 2.8 | 15 | 26.8 |
| 42003478100 | 1 | -79.98125826 | 40.37300584 | 0.587 | 0.08883 | 15.13 | 0.413 | 0.761 | 0.4767 | 0.7638 | 80.7 | 10.4 | 6.4 | 8 | 15.2 |
| 42003478200 | 1 | -79.9720812 | 40.3736181 | 0.627 | 0.09365 | 14.94 | 0.443 | 0.811 | 0.3956 | 0.6045 | 76.1 | 7.8 | 6.1 | 8 | 11 |
| 42003479000 | 0 | -80.01429369 | 40.38014668 | 0.471 | 0.12048 | 25.58 | 0.235 | 0.707 | 0.3872 | 0.3911 | 81.7 | 9.2 | 7.5 | 5.4 | 4.6 |
| 42003480101 | 1 | -79.98350412 | 40.34115581 | 0.519 | 0.08216 | 15.83 | 0.358 | 0.680 | 0.4272 | 0.8469 | 76.7 | 7.1 | 4.4 | 10.3 | 28.1 |
| 42003480102 | 1 | -79.99952821 | 40.34434092 | 0.465 | 0.07576 | 16.29 | 0.317 | 0.613 | 0.2901 | 0.4189 | 83.3 | 7.6 | 3.2 | 6.3 | 6.5 |
| 42003480200 | 1 | -79.96245502 | 40.35685294 | 0.373 | 0.10840 | 29.06 | 0.161 | 0.585 | 0.3433 | 0.6314 | 80.6 | 4.4 | 9.6 | 3.2 | 8.4 |
| 42003480300 | 0 | -79.96447229 | 40.3966739 | 0.464 | 0.12027 | 25.91 | 0.229 | 0.700 | 0.1496 | 0.0943 | 78.2 | 4.1 | 3.7 | 6.8 | 4.5 |
| 42003480400 | 1 | -79.95245653 | 40.38037304 | 0.423 | 0.07937 | 18.76 | 0.267 | 0.579 | 0.2704 | 0.3924 | 83.5 | 8.2 | 5.2 | 4.3 | 3.7 |
| 42003481000 | 1 | -79.98563559 | 40.41127311 | 0.581 | 0.08978 | 15.45 | 0.405 | 0.757 | 0.9183 | 0.8144 | 74.7 | 36.6 | 20.6 | 16.8 | 42.5 |
| 42003482500 | 0 | -79.91857645 | 40.39773793 | 0.466 | 0.12031 | 25.84 | 0.230 | 0.701 | 0.405 | 0.4449 | 84.8 | 10 | 6.3 | 4.7 | 13.3 |
| 42003483800 | 1 | -79.90971604 | 40.40731629 | 0.468 | 0.09950 | 21.26 | 0.273 | 0.663 | 0.7512 | 0.8547 | 76.1 | 29.1 | 6.6 | 8.8 | 74.3 |
| 42003484300 | 1 | -79.89643948 | 40.40620905 | 0.598 | 0.08438 | 14.11 | 0.433 | 0.763 | 0.6883 | 0.3905 | 78.9 | 15.8 | 10.9 | 7.6 | 33.9 |
| 42003484500 | 1 | -79.9042091 | 40.38220139 | 0.512 | 0.05692 | 11.12 | 0.400 | 0.624 | 0.43 | 0.4774 | 83.3 | 8.6 | 6.9 | 7.4 | 4 |
| 42003484600 | 0 | -79.90069318 | 40.39227769 | 0.469 | 0.12042 | 25.67 | 0.233 | 0.705 | 0.3205 | 0.308 | 74.9 | 7.7 | 4.5 | 5 | 17.5 |
| 42003485000 | 0 | -79.88635382 | 40.40069847 | 0.547 | 0.11983 | 21.91 | 0.312 | 0.782 | 0.5609 | 0.2309 | 82 | 13.2 | 5 | 8.6 | 11.5 |
| 42003486700 | 1 | -79.85382235 | 40.37935911 | 0.542 | 0.08432 | 15.56 | 0.377 | 0.707 | 0.8319 | 0.8544 | 73.1 | 47.7 | 23.6 | 12 | 65 |
| 42003486800 | 1 | -79.84477948 | 40.36802017 | 0.550 | 0.09721 | 17.67 | 0.359 | 0.741 | 0.8451 | 0.7666 | 69.9 | 30.3 | 27.7 | 7.5 | 75.1 |
| 42003486900 | 1 | -79.84816649 | 40.36412172 | 0.582 | 0.10913 | 18.75 | 0.368 | 0.796 | 0.933 | 0.4614 | 63.5 | 41.2 | 22.2 | 13.2 | 74.6 |
| 42003487000 | 1 | -79.89061854 | 40.35083652 | 0.710 | 0.07409 | 10.44 | 0.565 | 0.855 | 0.4279 | 0.0993 | 87.5 | 15.9 | 4.9 | 5.5 | 4.7 |
| 42003488100 | 1 | -79.88768279 | 40.38674116 | 0.478 | 0.09889 | 20.69 | 0.284 | 0.672 | 0.5834 | 0.5358 | 81 | 12.8 | 8.4 | 8.4 | 20.2 |
| 42003488200 | 0 | -79.87330063 | 40.3910543 | 0.586 | 0.11731 | 20.02 | 0.356 | 0.816 | 0.8044 | 0.3602 | 75.9 | 19.2 | 15.3 | 8.9 | 26 |
| 42003488300 | 1 | -79.88768964 | 40.37017739 | 0.514 | 0.09121 | 17.75 | 0.335 | 0.693 | 0.1186 | 0.0353 | 81.9 | 7 | 4.3 | 2.1 | 2.1 |
| 42003488400 | 1 | -79.86649463 | 40.36349415 | 0.532 | 0.07510 | 14.12 | 0.385 | 0.679 | 0.5202 | 0.279 | 86.3 | 9.1 | 5.7 | 9.7 | 15.3 |
| 42003488500 | 1 | -79.93398269 | 40.35885761 | 0.550 | 0.10464 | 19.03 | 0.345 | 0.755 | 0.3897 | 0.1884 | 82.1 | 10.5 | 7.3 | 1.8 | 8.7 |
| 42003488600 | 1 | -79.92037581 | 40.33796671 | 0.416 | 0.10954 | 26.33 | 0.201 | 0.631 | 0.4369 | 0.3718 | 81.4 | 16 | 8 | 2.5 | 12.5 |
| 42003489001 | 1 | -79.95070199 | 40.32949386 | 0.372 | 0.09524 | 25.60 | 0.185 | 0.559 | 0.266 | 0.4561 | 82.7 | 4.3 | 8.6 | 3.5 | 3.6 |
| 42003489002 | 1 | -79.96924526 | 40.33016342 | 0.372 | 0.09022 | 24.25 | 0.195 | 0.549 | 0.2507 | 0.4058 | 79.4 | 5 | 8.1 | 2.9 | 5.7 |
| 42003490002 | 1 | -80.01077224 | 40.29661876 | 0.467 | 0.09859 | 21.11 | 0.274 | 0.660 | 0.3393 | 0.5958 | 79 | 9 | 6.5 | 3.1 | 16.6 |
| 42003490003 | 0 | -79.99189099 | 40.31639913 | 0.366 | 0.11220 | 30.66 | 0.146 | 0.586 | 0.148 | 0.632 | 81.1 | 6.9 | 4 | 3.2 | 7.6 |
| 42003490004 | 1 | -79.98017199 | 40.28436465 | 0.467 | 0.09508 | 20.36 | 0.281 | 0.653 | 0.1693 | 0.3755 | 78.1 | 4.4 | 5.8 | 2.6 | 6.2 |
| 42003491101 | 1 | -79.95203725 | 40.30085797 | 0.417 | 0.04324 | 10.37 | 0.332 | 0.502 | 0.0313 | 0.3112 | 74.8 | 3 | 3.7 | 1.9 | 5.7 |
| 42003491200 | 0 | -79.91679988 | 40.31012083 | 0.466 | 0.12031 | 25.84 | 0.230 | 0.701 | 0.1349 | 0.0662 | 86.8 | 2.9 | 5.8 | 3.2 | 3.4 |
| 42003492700 | 1 | -79.88305752 | 40.30325907 | 0.581 | 0.11389 | 19.60 | 0.358 | 0.804 | 0.8623 | 0.727 | 78.5 | 30.8 | 11.8 | 13.7 | 25.8 |
| 42003492800 | 1 | -79.88290483 | 40.29201652 | 0.720 | 0.07714 | 10.71 | 0.569 | 0.871 | 0.7878 | 0.4536 | 83.1 | 36.4 | 20.2 | 4.4 | 67.9 |
| 42003492900 | 0 | -79.89383615 | 40.28799838 | 0.580 | 0.11782 | 20.33 | 0.349 | 0.811 | 0.8006 | 0.3896 | 77.9 | 23.2 | 10.8 | 9.1 | 33.6 |
| 42003494000 | 0 | -79.887371 | 40.271388 | 0.600 | 0.11605 | 19.34 | 0.373 | 0.827 | 0.6917 | 0.125 | 79.2 | 17.3 | 7.2 | 9.8 | 5.7 |
| 42003495000 | 1 | -79.90205092 | 40.22617105 | 0.512 | 0.10005 | 19.54 | 0.316 | 0.708 | 0.411 | 0.0197 | 84.8 | 3.1 | 9.7 | 7.8 | 1.3 |
| 42003496101 | 1 | -79.81913852 | 40.27060585 | 0.575 | 0.08712 | 15.15 | 0.404 | 0.746 | 0.3321 | 0.0047 | 79.4 | 5 | 9.2 | 3.9 | 0.3 |
| 42003496102 | 1 | -79.83879323 | 40.24530422 | 0.526 | 0.08562 | 16.28 | 0.358 | 0.694 | 0.2438 | 0.0637 | 82 | 6.7 | 5.4 | 3.8 | 3.2 |
| 42003496200 | 1 | -79.81000511 | 40.29829448 | 0.398 | 0.06768 | 17.00 | 0.265 | 0.531 | 0.3828 | 0.05 | 81.5 | 11.2 | 5.7 | 4.2 | 2.7 |
| 42003497000 | 1 | -79.85542107 | 40.2994779 | 0.491 | 0.11675 | 23.78 | 0.262 | 0.720 | 0.4689 | 0.0947 | 83.5 | 8.6 | 7.6 | 6.4 | 4.5 |
| 42003498000 | 1 | -79.85894274 | 40.32436839 | 0.603 | 0.09930 | 16.47 | 0.408 | 0.798 | 0.4567 | 0.3321 | 83.9 | 9.1 | 8.9 | 3.2 | 7 |
| 42003499300 | 1 | -79.88654184 | 40.33175182 | 0.527 | 0.10213 | 19.38 | 0.327 | 0.727 | 0.7111 | 0.1659 | 80.2 | 12.7 | 8.2 | 10 | 1 |
| 42003499400 | 1 | -79.8858353 | 40.32024384 | 0.576 | 0.08556 | 14.85 | 0.408 | 0.744 | 0.7853 | 0.4508 | 77.1 | 15.2 | 11 | 10.6 | 6.1 |
| 42003500300 | 1 | -79.87199533 | 40.33711774 | 0.650 | 0.07596 | 11.69 | 0.501 | 0.799 | 0.543 | 0.1656 | 81.6 | 10.5 | 5.6 | 9.8 | 7.8 |
| 42003501000 | 1 | -79.83084922 | 40.31772857 | 0.622 | 0.11023 | 17.72 | 0.406 | 0.838 | 0.77 | 0.3471 | 83.3 | 16.4 | 11.9 | 10.3 | 8 |
| 42003503002 | 1 | -79.81542599 | 40.34774998 | 0.482 | 0.05158 | 10.70 | 0.381 | 0.583 | 0.1465 | 0.4339 | 86.3 | 3.3 | 3.1 | 4.9 | 5.5 |
| 42003504100 | 1 | -79.82903557 | 40.38310291 | 0.510 | 0.07280 | 14.27 | 0.367 | 0.653 | 0.4908 | 0.3914 | 74.8 | 26.1 | 2.5 | 5.8 | 34 |
| 42003507000 | 1 | -79.80741385 | 40.38481541 | 0.595 | 0.09975 | 16.76 | 0.399 | 0.791 | 0.4789 | 0.4264 | 79.5 | 9.6 | 7.4 | 4.6 | 14.7 |
| 42003508000 | 1 | -79.81002212 | 40.39416981 | 0.486 | 0.11679 | 24.03 | 0.257 | 0.715 | 0.8754 | 0.692 | 77.1 | 28.8 | 27.7 | 10.5 | 35.6 |
| 42003509400 | 1 | -79.82146381 | 40.40857559 | 0.514 | 0.06301 | 12.26 | 0.391 | 0.637 | 0.5634 | 0.3605 | 76.1 | 24 | 4.7 | 5 | 26 |
| 42003510000 | 1 | -79.8381815 | 40.39687788 | 0.468 | 0.11606 | 24.80 | 0.241 | 0.695 | 0.811 | 0.7085 | 71.4 | 26.3 | 7.9 | 10.4 | 61.1 |
| 42003512000 | 1 | -79.84511046 | 40.40477917 | 0.496 | 0.10334 | 20.84 | 0.293 | 0.699 | 0.6191 | 0.7491 | 78.3 | 19.3 | 5.6 | 7.5 | 37.1 |
| 42003512800 | 1 | -79.85547012 | 40.39920953 | 0.364 | 0.09586 | 26.34 | 0.176 | 0.552 | 0.923 | 0.8232 | 66.7 | 54.9 | 13.2 | 14.1 | 66.2 |
| 42003512900 | 0 | -79.8647705 | 40.40964658 | 0.587 | 0.11720 | 19.95 | 0.358 | 0.817 | 0.8748 | 0.4511 | 78.2 | 27.5 | 13.6 | 13.9 | 65.6 |
| 42003513800 | 1 | -79.86883695 | 40.40189758 | 0.450 | 0.08426 | 18.72 | 0.285 | 0.615 | 0.9277 | 0.7769 | 81.9 | 30.3 | 16.9 | 16.1 | 70.8 |
| 42003514000 | 1 | -79.8811283 | 40.41099959 | 0.604 | 0.09476 | 15.69 | 0.418 | 0.790 | 0.9192 | 0.7226 | 70.2 | 38 | 17.9 | 14.1 | 81 |
| 42003515100 | 1 | -79.87856245 | 40.41825802 | 0.608 | 0.08264 | 13.59 | 0.446 | 0.770 | 0.7136 | 0.4386 | 80.2 | 22.2 | 12.8 | 3.7 | 58 |
| 42003515200 | 1 | -79.87958754 | 40.42491365 | 0.507 | 0.07197 | 14.20 | 0.366 | 0.648 | 0.3549 | 0.3918 | 84.1 | 5.9 | 9.4 | 1.9 | 34.1 |
| 42003515300 | 1 | -79.89215609 | 40.41775698 | 0.434 | 0.10719 | 24.70 | 0.224 | 0.644 | 0.8444 | 0.8694 | 85.9 | 25.8 | 12.8 | 11.9 | 46.2 |
| 42003515401 | 0 | -79.89420694 | 40.42600058 | 0.409 | 0.11690 | 28.57 | 0.180 | 0.638 | 0.297 | 0.5986 | 85 | 17.7 | 4.4 | 3.7 | 18.5 |
| 42003516100 | 1 | -79.88860542 | 40.43091833 | 0.293 | 0.09327 | 31.83 | 0.110 | 0.476 | 0.0958 | 0.6311 | 82.3 | 9.1 | 3.9 | 1.4 | 11.7 |
| 42003516200 | 1 | -79.87987593 | 40.43163088 | 0.380 | 0.10493 | 27.61 | 0.174 | 0.586 | 0.1568 | 0.7154 | 80 | 11.2 | 4.1 | 2.4 | 22.9 |
| 42003517000 | 0 | -79.8629284 | 40.41800932 | 0.461 | 0.12014 | 26.08 | 0.225 | 0.696 | 0.5452 | 0.6676 | 86.9 | 18.5 | 6 | 7.4 | 36.6 |
| 42003518001 | 1 | -79.85923393 | 40.4281526 | 0.423 | 0.05899 | 13.95 | 0.307 | 0.539 | 0.026 | 0.497 | 86.6 | 3.9 | 3.3 | 0.6 | 7.9 |
| 42003519000 | 0 | -79.84160027 | 40.43885026 | 0.345 | 0.10923 | 31.69 | 0.131 | 0.559 | 0.0739 | 0.6514 | 84.5 | 2.7 | 6 | 0.4 | 24.5 |
| 42003520001 | 0 | -79.81613498 | 40.43108494 | 0.432 | 0.11868 | 27.44 | 0.200 | 0.665 | 0.3105 | 0.4905 | 83.3 | 9.8 | 6.5 | 4 | 17.4 |
| 42003520002 | 1 | -79.83405902 | 40.41908543 | 0.400 | 0.11082 | 27.70 | 0.183 | 0.617 | 0.379 | 0.5139 | 82.7 | 6.6 | 10.2 | 2.7 | 21.5 |
| 42003521100 | 1 | -79.77678214 | 40.45136683 | 0.330 | 0.06731 | 20.40 | 0.198 | 0.462 | 0.1715 | 0.8022 | 81.9 | 7.5 | 3.3 | 4 | 30.4 |
| 42003521200 | 1 | -79.79654469 | 40.42571892 | 0.437 | 0.09612 | 22.00 | 0.249 | 0.625 | 0.3621 | 0.7382 | 84 | 8.1 | 7.2 | 4.8 | 23.3 |
| 42003521301 | 1 | -79.76334022 | 40.41661966 | 0.361 | 0.08521 | 23.60 | 0.194 | 0.528 | 0.3524 | 0.6173 | 82.2 | 16.3 | 3.6 | 6.4 | 35.3 |
| 42003521302 | 1 | -79.78592354 | 40.41530536 | 0.532 | 0.06979 | 13.12 | 0.395 | 0.669 | 0.2829 | 0.6667 | 82 | 7.6 | 4.6 | 6 | 23.9 |
| 42003521401 | 1 | -79.73195307 | 40.42472738 | 0.457 | 0.07176 | 15.70 | 0.316 | 0.598 | 0.1581 | 0.5701 | 87.1 | 4.9 | 2.8 | 6.5 | 20.2 |
| 42003521402 | 1 | -79.74727342 | 40.40387224 | 0.563 | 0.06565 | 11.66 | 0.434 | 0.692 | 0.0667 | 0.6598 | 81.2 | 3.4 | 4.6 | 2.1 | 21.1 |
| 42003521500 | 0 | -79.73383771 | 40.44352229 | 0.394 | 0.11541 | 29.32 | 0.167 | 0.620 | 0.2385 | 0.6029 | 82.3 | 9.4 | 4.7 | 4.8 | 13.6 |
| 42003522000 | 1 | -79.77634397 | 40.40777955 | 0.618 | 0.09905 | 16.03 | 0.424 | 0.812 | 0.7881 | 0.3683 | 78.7 | 21.4 | 11.5 | 9 | 27.9 |
| 42003523100 | 1 | -79.87106834 | 40.47304155 | 0.558 | 0.08491 | 15.22 | 0.392 | 0.724 | 0.7609 | 0.7532 | 82 | 16.8 | 12.5 | 9.7 | 87.8 |
| 42003523200 | 1 | -79.85336991 | 40.46261448 | 0.446 | 0.09028 | 20.24 | 0.269 | 0.623 | 0.6482 | 0.8132 | 75 | 18.9 | 12.9 | 4.7 | 69.4 |
| 42003523300 | 1 | -79.83437737 | 40.47271828 | 0.561 | 0.08000 | 14.26 | 0.404 | 0.718 | 0.5546 | 0.6642 | 80.3 | 9.5 | 12.5 | 5.3 | 42.5 |
| 42003523400 | 1 | -79.83347312 | 40.45618983 | 0.549 | 0.05857 | 10.67 | 0.434 | 0.664 | 0.4182 | 0.4183 | 77.8 | 13.6 | 5.1 | 4.5 | 44.4 |
| 42003523501 | 1 | -79.81049349 | 40.47230356 | 0.328 | 0.08643 | 26.35 | 0.159 | 0.497 | 0.4423 | 0.7638 | 82.6 | 6.9 | 9.3 | 4.7 | 53.1 |
| 42003523502 | 1 | -79.79014163 | 40.46419287 | 0.433 | 0.11127 | 25.70 | 0.215 | 0.651 | 0.7186 | 0.6567 | 81.8 | 17 | 7.3 | 9.5 | 28.3 |
| 42003523600 | 1 | -79.81235941 | 40.45095093 | 0.308 | 0.05727 | 18.59 | 0.196 | 0.420 | 0.3706 | 0.6129 | 82.3 | 11 | 5.2 | 6.6 | 38.5 |
| 42003523701 | 1 | -79.81813863 | 40.50410858 | 0.592 | 0.07266 | 12.27 | 0.450 | 0.734 | 0.5064 | 0.4855 | 82.3 | 9.8 | 6.6 | 9.9 | 18.7 |
| 42003523702 | 0 | -79.79768322 | 40.49036457 | 0.445 | 0.11942 | 26.84 | 0.211 | 0.679 | 0.4319 | 0.5929 | 83.3 | 10.1 | 6.4 | 5.9 | 28.6 |
| 42003523800 | 1 | -79.83946858 | 40.4885084 | 0.537 | 0.06427 | 11.97 | 0.411 | 0.663 | 0.2304 | 0.5645 | 85.9 | 3.2 | 3.8 | 8.8 | 14.3 |
| 42003524000 | 0 | -79.84187384 | 40.50503209 | 0.506 | 0.12087 | 23.88 | 0.269 | 0.743 | 0.693 | 0.6332 | 79.9 | 22 | 8 | 6.9 | 15.5 |
| 42003525100 | 1 | -79.82924499 | 40.51719741 | 0.388 | 0.07823 | 20.16 | 0.235 | 0.541 | 0.0085 | 0.1428 | 83.5 | 2.3 | 1.3 | 2.5 | 6.6 |
| 42003525200 | 1 | -79.8449182 | 40.51617584 | 0.515 | 0.09455 | 18.36 | 0.330 | 0.700 | 0.3174 | 0.0066 | 89.1 | 8.2 | 4.2 | 8.8 | 0.5 |
| 42003525300 | 1 | -79.83537626 | 40.52513345 | 0.561 | 0.11167 | 19.91 | 0.342 | 0.780 | 0.2498 | 0.13 | 80.3 | 7.6 | 6.1 | 5.7 | 5.9 |
| 42003526101 | 0 | -79.76654801 | 40.52375776 | 0.378 | 0.11365 | 30.09 | 0.155 | 0.600 | 0.0385 | 0.4117 | 81.2 | 3.3 | 1.2 | 4.5 | 6.9 |
| 42003526102 | 0 | -79.72160176 | 40.50211323 | 0.493 | 0.12086 | 24.49 | 0.257 | 0.730 | 0.3121 | 0.1665 | 78 | 4.9 | 6.1 | 7.5 | 0.6 |
| 42003526201 | 0 | -79.75777989 | 40.48881634 | 0.438 | 0.11900 | 27.20 | 0.204 | 0.671 | 0.2826 | 0.4239 | 78.7 | 4.2 | 7.7 | 4.8 | 5.9 |
| 42003526202 | 0 | -79.75208442 | 40.46822035 | 0.510 | 0.12084 | 23.69 | 0.273 | 0.747 | 0.4144 | 0.2215 | 77.2 | 5.4 | 7.5 | 7.9 | 10.8 |
| 42003526301 | 0 | -79.71844257 | 40.47602486 | 0.453 | 0.11982 | 26.44 | 0.218 | 0.688 | 0.2451 | 0.2874 | 76.7 | 7.2 | 5.1 | 2.8 | 2.8 |
| 42003526302 | 1 | -79.71060389 | 40.46182754 | 0.468 | 0.06797 | 14.52 | 0.335 | 0.601 | 0.1049 | 0.0843 | 79.1 | 3.6 | 3.1 | 3.5 | 4.1 |
| 42003550900 | 1 | -79.8518338 | 40.3484402 | 0.607 | 0.11229 | 18.50 | 0.387 | 0.827 | 0.949 | 0.6935 | 78.8 | 38.1 | 22.8 | 17.2 | 56.5 |
| 42003551200 | 1 | -79.83560993 | 40.35091003 | 0.494 | 0.08866 | 17.95 | 0.320 | 0.668 | 0.9167 | 0.7332 | 75.2 | 41.5 | 17.7 | 14 | 40.7 |
| 42003551300 | 0 | -79.82734035 | 40.33246495 | 0.468 | 0.12039 | 25.73 | 0.232 | 0.704 | 0.3609 | 0.3708 | 86.2 | 14 | 3.9 | 5.9 | 7.2 |
| 42003551900 | 0 | -79.85027496 | 40.35520417 | 0.560 | 0.11912 | 21.25 | 0.327 | 0.794 | 0.9593 | 0.7157 | 84.4 | 56.2 | 20.6 | 15.9 | 48.4 |
| 42003552000 | 0 | -79.84090556 | 40.34156331 | 0.596 | 0.11643 | 19.54 | 0.368 | 0.824 | 0.8995 | 0.4383 | 73.9 | 23.3 | 21.8 | 16.4 | 58 |
| 42003552100 | 0 | -79.86365941 | 40.34518022 | 0.568 | 0.11868 | 20.91 | 0.335 | 0.800 | 0.7934 | 0.4449 | 80.3 | 72.8 | 0.8 | 25.4 | 62.2 |
| 42003552200 | 0 | -79.8772478 | 40.34915811 | 0.604 | 0.11569 | 19.16 | 0.377 | 0.830 | 0.8432 | 0.3168 | 88.3 | 23.6 | 10.1 | 13.5 | 18.6 |
| 42003552300 | 0 | -79.8513514 | 40.34175414 | 0.592 | 0.11679 | 19.73 | 0.363 | 0.821 | 0.8973 | 0.457 | 72.3 | 42.9 | 25.1 | 10.6 | 71.1 |
| 42003552400 | 0 | -79.84433275 | 40.3329505 | 0.589 | 0.11704 | 19.87 | 0.360 | 0.819 | 0.7759 | 0.3027 | 79.9 | 15.8 | 9.7 | 11.6 | 17.1 |
| 42003560400 | 0 | -79.89024686 | 40.44466424 | 0.586 | 0.11731 | 20.02 | 0.356 | 0.816 | 0.8695 | 0.4514 | 87.7 | 30.6 | 11.9 | 14.6 | 66.4 |
| 42003560500 | 1 | -79.89127175 | 40.43777388 | 0.389 | 0.06964 | 17.90 | 0.253 | 0.525 | 0.2808 | 0.5489 | 90.4 | 9.8 | 7.6 | 2.9 | 16.9 |
| 42003560600 | 0 | -79.88381083 | 40.44039436 | 0.589 | 0.11710 | 19.90 | 0.359 | 0.818 | 0.8895 | 0.4652 | 78.7 | 23.4 | 22.3 | 14.2 | 77.8 |
| 42003561000 | 0 | -79.87567803 | 40.43649591 | 0.530 | 0.12044 | 22.71 | 0.294 | 0.766 | 0.9023 | 0.7976 | 70.3 | 33.1 | 15.3 | 14.4 | 83.3 |
| 42003561100 | 0 | -79.87584537 | 40.44663678 | 0.592 | 0.11678 | 19.72 | 0.363 | 0.821 | 0.9296 | 0.5017 | 72.3 | 40 | 15.1 | 15.2 | 96 |
| 42003561200 | 0 | -79.88241736 | 40.44617593 | 0.520 | 0.12070 | 23.22 | 0.283 | 0.756 | 0.8842 | 0.8282 | 74.6 | 37.2 | 12.8 | 12.3 | 94.1 |
| 42003561400 | 1 | -79.86202048 | 40.45064216 | 0.469 | 0.08532 | 18.19 | 0.302 | 0.636 | 0.6933 | 0.6885 | 87.4 | 15.9 | 15.1 | 7.8 | 80.3 |
| 42003561500 | 1 | -79.86841572 | 40.44031432 | 0.539 | 0.11283 | 20.93 | 0.318 | 0.760 | 0.7812 | 0.6992 | 84.9 | 28.8 | 12.3 | 7.5 | 75 |
| 42003561600 | 1 | -79.96635111 | 40.41484061 | 0.658 | 0.09132 | 13.88 | 0.479 | 0.837 | 0.8019 | 0.5967 | 80.2 | 19.4 | 6.7 | 15.7 | 33.2 |
| 42003561700 | 0 | -79.97400674 | 40.40932081 | 0.492 | 0.12085 | 24.57 | 0.255 | 0.729 | 0.8538 | 0.9335 | 70.2 | 13.9 | 10.9 | 19.6 | 64.5 |
| 42003561900 | 0 | -79.89308742 | 40.46935932 | 0.586 | 0.11733 | 20.03 | 0.356 | 0.816 | 0.8886 | 0.4789 | 83.4 | 29 | 19.2 | 15 | 87.3 |
| 42003562000 | 1 | -79.95389795 | 40.45183578 | 0.379 | 0.09338 | 24.64 | 0.196 | 0.562 | 0.7737 | 0.8763 | 95.7 | 44.9 | 11.8 | 7.7 | 46.4 |
| 42003562300 | 1 | -79.93273311 | 40.40865027 | 0.653 | 0.09747 | 14.93 | 0.462 | 0.844 | 0.8485 | 0.6932 | 74.9 | 43.2 | 8.9 | 10.6 | 61.8 |
| 42003562400 | 1 | -80.00243267 | 40.41271468 | 0.468 | 0.11054 | 23.62 | 0.251 | 0.685 | 0.6839 | 0.865 | 83.6 | 16 | 9.3 | 9.3 | 65.7 |
| 42003562500 | 1 | -80.05241848 | 40.45789178 | 0.429 | 0.11216 | 26.14 | 0.209 | 0.649 | 0.8579 | 0.8775 | 73.8 | 33 | 16.3 | 9.8 | 62.7 |
| 42003562600 | 0 | -80.03689096 | 40.44318179 | 0.581 | 0.11774 | 20.28 | 0.350 | 0.811 | 0.8119 | 0.3996 | 73.3 | 25.8 | 12.7 | 8.1 | 37.4 |
| 42003562700 | 1 | -80.00986869 | 40.45194042 | 0.357 | 0.10188 | 28.54 | 0.157 | 0.557 | 0.4811 | 0.7101 | 92 | 25.6 | 5.4 | 8.4 | 34.6 |
| 42003562800 | 0 | -80.07135715 | 40.42269616 | 0.497 | 0.12088 | 24.31 | 0.260 | 0.734 | 0.7121 | 0.7067 | 85.4 | 19.7 | 7.4 | 10.9 | 15.7 |
| 42003562900 | 0 | -79.94721657 | 40.40029213 | 0.548 | 0.11975 | 21.84 | 0.314 | 0.783 | 0.667 | 0.3711 | 78.5 | 20.8 | 8 | 6.2 | 28.8 |
| 42003563000 | 1 | -80.08092176 | 40.45287843 | 0.438 | 0.11118 | 25.38 | 0.220 | 0.656 | 0.569 | 0.4117 | 85.3 | 18 | 7 | 7.3 | 42.3 |
| 42003563100 | 1 | -80.04371885 | 40.43092976 | 0.449 | 0.10733 | 23.90 | 0.239 | 0.659 | 0.4798 | 0.8738 | 78 | 12.7 | 2.1 | 13.1 | 25.1 |
| 42003563200 | 1 | -79.99793124 | 40.45292564 | 0.424 | 0.08922 | 21.04 | 0.249 | 0.599 | 0.6197 | 0.6757 | 93 | 25.4 | 6.1 | 13.6 | 27.9 |
| 42003563300 | 0 | -80.14631826 | 40.5616318 | 0.347 | 0.10963 | 31.55 | 0.133 | 0.562 | 0.0041 | 0.5376 | 75 | 3.7 | 1.7 | 1.1 | 10.2 |
| 42003563800 | 1 | -80.12410383 | 40.52488448 | 0.404 | 0.10436 | 25.83 | 0.199 | 0.609 | 0.0291 | 0.2477 | 81.7 | 4.1 | 2.7 | 2.7 | 5.4 |
| 42003563900 | 1 | -79.84456412 | 40.41767462 | 0.616 | 0.07969 | 12.94 | 0.460 | 0.772 | 0.3218 | 0.3533 | 83.8 | 10.3 | 5.8 | 3.6 | 24.9 |
| 42003564000 | 1 | -80.24024874 | 40.41290349 | 0.335 | 0.08983 | 26.82 | 0.159 | 0.511 | 0.2911 | 0.3864 | 77 | 6.7 | 4.2 | 11.4 | 4 |
| 42003564100 | 0 | -80.08662507 | 40.42771352 | 0.403 | 0.11636 | 28.86 | 0.175 | 0.631 | 0.0006 | 0.2162 | 79.4 | 0.9 | 2.1 | 0.1 | 3.9 |
| 42003564200 | 1 | -79.7942185 | 40.33255602 | 0.493 | 0.11524 | 23.38 | 0.267 | 0.719 | 0.2466 | 0.393 | 85.9 | 6.1 | 6.2 | 6.6 | 7.4 |
| 42003564400 | 1 | -79.79129344 | 40.37647313 | 0.497 | 0.09375 | 18.86 | 0.313 | 0.681 | 0.4773 | 0.3939 | 86.7 | 9.8 | 7.8 | 6.5 | 9.6 |
| 42003564500 | 1 | -79.92240879 | 40.27322989 | 0.438 | 0.10597 | 24.19 | 0.230 | 0.646 | 0.2654 | 0.3358 | 78.3 | 5.8 | 5.7 | 5.8 | 3.2 |
| 42003980000 | 0 | -79.8924051 | 40.48825426 | 0.383 | 0.11424 | 29.85 | 0.159 | 0.607 | 0.0207 | 0.3583 | 91.4 | 7 | 0 | 0 | 25.7 |
| 42003980500 | 0 | -79.94215637 | 40.43434547 | 0.495 | 0.12087 | 24.40 | 0.258 | 0.732 | 0.2006 | 0 | 100 | 0 | 0 | 23.5 | 0 |
| 42003981800 | 0 | -79.89721704 | 40.48017061 | 0.509 | 0.12084 | 23.72 | 0.273 | 0.746 | 0.805 | 0.7726 | 84.2 | 72.4 | 23.1 | 0 | 46.1 |
| 42003982200 | 1 | -79.95476545 | 40.44448802 | 0.502 | 0.10276 | 20.47 | 0.301 | 0.703 | 0.9894 | 0.442 | 98.2 | 80.3 | 21.4 | 23.8 | 17 |
| 42007601800 | 1 | -80.19791711 | 40.72148614 | 0.402 | 0.10498 | 26.11 | 0.196 | 0.608 | 0.2917 | 0.0075 | 81.4 | 7.7 | 4 | 8.2 | 0.6 |
| 42007603700 | 0 | -80.2228358 | 40.63966211 | 0.472 | 0.12049 | 25.55 | 0.235 | 0.708 | 0.3618 | 0.3527 | 86 | 8.9 | 5.3 | 7.2 | 6.2 |
| 42007603801 | 0 | -80.20998732 | 40.66182507 | 0.456 | 0.11997 | 26.28 | 0.221 | 0.692 | 0.0826 | 0.0419 | 83.6 | 3.9 | 3.2 | 4.1 | 2.4 |
| 42007603802 | 0 | -80.16360132 | 40.65773859 | 0.457 | 0.12000 | 26.25 | 0.222 | 0.692 | 0.0823 | 0.0378 | 84 | 3 | 4.6 | 4.2 | 2.2 |
| 42007603803 | 0 | -80.18111048 | 40.62585776 | 0.407 | 0.11667 | 28.69 | 0.178 | 0.635 | 0.1305 | 0.3796 | 82 | 4.5 | 2.8 | 6.5 | 3 |
| 42007603900 | 0 | -80.21887459 | 40.60867706 | 0.467 | 0.12037 | 25.76 | 0.231 | 0.703 | 0.2983 | 0.2865 | 80.4 | 7.5 | 5.5 | 4.3 | 2.2 |
| 42007604000 | 0 | -80.22028105 | 40.60026935 | 0.592 | 0.11682 | 19.74 | 0.363 | 0.821 | 0.8382 | 0.3758 | 77.8 | 21.1 | 12.5 | 11.8 | 30.4 |
| 42007604100 | 1 | -80.2275035 | 40.58309107 | 0.620 | 0.11041 | 17.81 | 0.404 | 0.836 | 0.7837 | 0.378 | 78.4 | 18 | 7.9 | 11.6 | 30.8 |
| 42007604200 | 0 | -80.23083705 | 40.59676267 | 0.547 | 0.11983 | 21.92 | 0.312 | 0.781 | 0.7872 | 0.5489 | 79.1 | 19.9 | 11.7 | 9.8 | 21.6 |
| 42007604901 | 0 | -80.28241673 | 40.59919892 | 0.403 | 0.11636 | 28.86 | 0.175 | 0.631 | 0.2961 | 0.6301 | 81.8 | 6.2 | 3.3 | 9.6 | 11.8 |
| 42007605001 | 0 | -80.33233498 | 40.54210477 | 0.468 | 0.12038 | 25.73 | 0.232 | 0.704 | 0.3083 | 0.2977 | 79.7 | 9.3 | 5 | 5.1 | 1.8 |
| 42007605002 | 1 | -80.44287219 | 40.51791961 | 0.467 | 0.11212 | 24.01 | 0.247 | 0.687 | 0.4225 | 0.3402 | 79.3 | 6.4 | 9.3 | 6.7 | 5.6 |
| 42019912002 | 0 | -80.12647344 | 40.70387742 | 0.387 | 0.11472 | 29.64 | 0.162 | 0.612 | 0.1086 | 0.4574 | 76.3 | 2.3 | 5.4 | 5.3 | 12.8 |
| 42019912200 | 0 | -80.12004285 | 40.67849073 | 0.395 | 0.11555 | 29.26 | 0.168 | 0.621 | 0.0401 | 0.3174 | 81.6 | 2.3 | 2.8 | 4.4 | 7.5 |
| 42019912303 | 0 | -80.0639327 | 40.68669307 | 0.355 | 0.11074 | 31.18 | 0.138 | 0.572 | 0.0013 | 0.4886 | 74.5 | 0.8 | 2 | 0.7 | 10.8 |
| 42019912304 | 0 | -80.081492 | 40.69110496 | 0.445 | 0.11940 | 26.85 | 0.211 | 0.679 | 0.02 | 0.0178 | 72.7 | 0.8 | 3.8 | 1.5 | 1.3 |
| 42019912401 | 1 | -80.03678182 | 40.70316328 | 0.320 | 0.06277 | 19.62 | 0.197 | 0.443 | 0.0063 | 0.1265 | 72.9 | 1.2 | 2.4 | 2.4 | 5.7 |
| 42019912402 | 1 | -79.99480249 | 40.7135545 | 0.480 | 0.11000 | 22.92 | 0.264 | 0.696 | 0.1108 | 0.2905 | 80.1 | 4.7 | 4.6 | 4.4 | 4.9 |
| 42019912800 | 1 | -79.92171075 | 40.70886066 | 0.418 | 0.09925 | 23.74 | 0.223 | 0.613 | 0.057 | 0.1062 | 76.1 | 1.9 | 2.5 | 3.1 | 0.4 |
| 42059970200 | 0 | -80.04972835 | 39.92305102 | 0.551 | 0.11964 | 21.73 | 0.316 | 0.785 | 0.4347 | 0.0331 | 83.9 | 8 | 5.7 | 9 | 2 |
| 42059970300 | 0 | -80.21353602 | 39.96639164 | 0.586 | 0.11730 | 20.02 | 0.356 | 0.816 | 0.5568 | 0.0125 | 81.9 | 11.1 | 5.5 | 11.9 | 1 |
| 42125711000 | 1 | -80.46109826 | 40.40035293 | 0.529 | 0.10747 | 20.32 | 0.318 | 0.740 | 0.4135 | 0.2321 | 82.2 | 6 | 5.3 | 10.8 | 2.6 |
| 42125713700 | 1 | -80.37168305 | 40.37417043 | 0.553 | 0.10986 | 19.87 | 0.338 | 0.768 | 0.6297 | 0.2284 | 80.4 | 9.9 | 6.2 | 15.6 | 0.6 |
| 42125714000 | 1 | -80.30724226 | 40.40914673 | 0.664 | 0.10085 | 15.19 | 0.466 | 0.862 | 0.6407 | 0.1043 | 80 | 11 | 8.3 | 9.7 | 4.9 |
| 42125715700 | 0 | -80.23585227 | 40.36913468 | 0.536 | 0.12025 | 22.43 | 0.301 | 0.772 | 0.6626 | 0.4299 | 82 | 11.5 | 6.9 | 11.6 | 5 |
| 42125722700 | 1 | -80.30552047 | 40.30403952 | 0.435 | 0.11467 | 26.36 | 0.210 | 0.660 | 0.2009 | 0.0506 | 78.7 | 4.3 | 3.9 | 8.1 | 2.7 |
| 42125741100 | 1 | -80.21985396 | 40.33425705 | 0.366 | 0.09006 | 24.61 | 0.189 | 0.543 | 0.1243 | 0.5948 | 78.6 | 2.6 | 3.6 | 7.2 | 10.3 |
| 42125741300 | 0 | -80.15107093 | 40.30363617 | 0.373 | 0.11307 | 30.33 | 0.151 | 0.594 | 0.1127 | 0.5433 | 83.7 | 3.6 | 4 | 5.3 | 5 |
| 42125746100 | 1 | -80.08669337 | 40.29331884 | 0.484 | 0.08689 | 17.95 | 0.314 | 0.654 | 0.0692 | 0.1181 | 76.5 | 2 | 5.6 | 2.2 | 5.5 |
| 42125746200 | 0 | -80.11572012 | 40.28452507 | 0.360 | 0.11142 | 30.94 | 0.142 | 0.578 | 0.0241 | 0.492 | 74.5 | 2.1 | 4.1 | 0.9 | 7 |
| 42125746302 | 0 | -80.04755835 | 40.26506756 | 0.423 | 0.11804 | 27.89 | 0.192 | 0.655 | 0.0069 | 0.115 | 64.3 | 2.1 | 2.8 | 0 | 5.4 |
| 42125761000 | 0 | -80.07568158 | 40.12444251 | 0.490 | 0.12084 | 24.66 | 0.253 | 0.727 | 0.3696 | 0.2652 | 81.9 | 7.1 | 6 | 8.3 | 2.6 |
| 42125762000 | 0 | -80.08808942 | 40.03267583 | 0.561 | 0.11907 | 21.21 | 0.328 | 0.795 | 0.5321 | 0.1122 | 79.3 | 16.9 | 5.3 | 10 | 5.3 |
| 42125771100 | 0 | -80.02501383 | 40.22374341 | 0.464 | 0.12027 | 25.91 | 0.229 | 0.700 | 0.231 | 0.2084 | 81.4 | 7.8 | 5.3 | 4.2 | 3 |
| 42125771200 | 1 | -80.00731966 | 40.26830393 | 0.594 | 0.10203 | 17.18 | 0.394 | 0.794 | 0.508 | 0.3458 | 84.8 | 10.3 | 7.3 | 7.8 | 3.1 |
| 42125772700 | 0 | -79.9531901 | 40.20638815 | 0.557 | 0.11931 | 21.42 | 0.323 | 0.791 | 0.7546 | 0.4474 | 80.7 | 14.8 | 9.9 | 12.7 | 8.1 |
| 42125773100 | 0 | -79.91992953 | 40.19466266 | 0.565 | 0.11883 | 21.03 | 0.332 | 0.798 | 0.8031 | 0.4714 | 77.5 | 17.4 | 9.6 | 12.9 | 10.4 |
| 42125773200 | 0 | -79.9368047 | 40.20221691 | 0.575 | 0.11813 | 20.53 | 0.344 | 0.807 | 0.5944 | 0.1231 | 85.1 | 14.5 | 10.2 | 5.2 | 5.6 |
| 42125774700 | 1 | -79.93122132 | 40.18128742 | 0.465 | 0.09513 | 20.46 | 0.279 | 0.651 | 0.1149 | 0.3155 | 83.2 | 5.6 | 1.3 | 4.8 | 6 |
| 42125781700 | 1 | -79.95666822 | 40.14237748 | 0.495 | 0.10733 | 21.68 | 0.285 | 0.705 | 0.3825 | 0.423 | 82.6 | 8.1 | 6 | 6.2 | 6.6 |
| 42125795700 | 0 | -80.016219 | 39.995013 | 0.647 | 0.11039 | 17.05 | 0.431 | 0.864 | 0.8316 | 0.0525 | 76.3 | 23 | 10.5 | 12.2 | 2.8 |
| 42125795800 | 0 | -80.19819276 | 40.29365799 | 0.444 | 0.11939 | 26.87 | 0.210 | 0.678 | 0.0726 | 0.0931 | 72.1 | 2.6 | 3.8 | 5.4 | 4.4 |
| 42129800100 | 0 | -79.77037452 | 40.57793494 | 0.623 | 0.11355 | 18.22 | 0.401 | 0.846 | 0.9609 | 0.3724 | 79.6 | 54.1 | 18.7 | 16.8 | 29.4 |
| 42129800200 | 0 | -79.76484535 | 40.57569555 | 0.521 | 0.12067 | 23.15 | 0.285 | 0.758 | 0.8739 | 0.8063 | 73 | 29.1 | 11.7 | 13.7 | 47.8 |
| 42129800300 | 0 | -79.76038721 | 40.58102996 | 0.526 | 0.12056 | 22.93 | 0.290 | 0.762 | 0.7365 | 0.5889 | 77.7 | 33.6 | 6.3 | 8.9 | 22.1 |
| 42129800400 | 0 | -79.75705983 | 40.59037006 | 0.463 | 0.12023 | 25.95 | 0.228 | 0.699 | 0.5906 | 0.7176 | 88 | 14.4 | 10.6 | 4.9 | 9.8 |
| 42129800500 | 0 | -79.74983404 | 40.57691549 | 0.439 | 0.11909 | 27.12 | 0.206 | 0.673 | 0.1199 | 0.1874 | 85.1 | 5.3 | 1.7 | 5.3 | 8.7 |
| 42129800600 | 0 | -79.76099259 | 40.57020249 | 0.552 | 0.11958 | 21.66 | 0.318 | 0.786 | 0.9083 | 0.6895 | 76.1 | 35.5 | 10.5 | 24.2 | 29 |
| 42129800700 | 0 | -79.76578506 | 40.56173019 | 0.625 | 0.11339 | 18.16 | 0.402 | 0.847 | 0.9706 | 0.3783 | 75.9 | 46.9 | 16.4 | 27.8 | 30.9 |
| 42129800800 | 0 | -79.75204408 | 40.55712258 | 0.509 | 0.12084 | 23.73 | 0.272 | 0.746 | 0.3759 | 0.1724 | 83 | 10.9 | 6.2 | 5.4 | 7.9 |
| 42129800900 | 0 | -79.7417985 | 40.56633298 | 0.525 | 0.12058 | 22.96 | 0.289 | 0.761 | 0.4739 | 0.2249 | 80.3 | 11.3 | 6.2 | 7.5 | 11.1 |
| 42129801001 | 1 | -79.7284732 | 40.58434439 | 0.488 | 0.11709 | 23.99 | 0.259 | 0.717 | 0.4 | 0.0169 | 82.1 | 7 | 8.1 | 5.7 | 1.2 |
| 42129801002 | 0 | -79.71052683 | 40.56973095 | 0.416 | 0.11748 | 28.24 | 0.186 | 0.646 | 0.389 | 0.6898 | 81.9 | 12.4 | 4.1 | 8.1 | 10.9 |
| 42129801100 | 1 | -79.71519845 | 40.59895203 | 0.606 | 0.10218 | 16.86 | 0.406 | 0.806 | 0.3308 | 0.0091 | 78.6 | 9.3 | 4.8 | 4.7 | 0.7 |
| 42129801200 | 1 | -79.64760154 | 40.60470248 | 0.483 | 0.08922 | 18.47 | 0.308 | 0.658 | 0.189 | 0.2802 | 79.5 | 6.6 | 3.4 | 6.1 | 3.8 |
| 42129801701 | 0 | -79.67286562 | 40.54719338 | 0.415 | 0.11741 | 28.27 | 0.185 | 0.645 | 0.1966 | 0.4246 | 82.5 | 4.6 | 6.1 | 3 | 5.6 |
| 42129801702 | 1 | -79.62087068 | 40.50879696 | 0.417 | 0.11278 | 27.05 | 0.196 | 0.638 | 0.2153 | 0.1818 | 81.4 | 3.4 | 4.7 | 7.4 | 2 |
| 42129802101 | 1 | -79.69068368 | 40.47176623 | 0.327 | 0.09803 | 29.98 | 0.135 | 0.519 | 0.0942 | 0.3799 | 82.4 | 3 | 4.8 | 4.6 | 3.4 |
| 42129802200 | 0 | -79.75558678 | 40.38491475 | 0.505 | 0.12087 | 23.92 | 0.268 | 0.742 | 0.2579 | 0.0275 | 80.8 | 13 | 3.4 | 4.4 | 1.7 |
| 42129802301 | 0 | -79.7072408 | 40.37985069 | 0.452 | 0.11976 | 26.51 | 0.217 | 0.686 | 0.0379 | 0.005 | 79 | 2.5 | 2.8 | 3.1 | 0.4 |
| 42129802304 | 1 | -79.65341136 | 40.3825466 | 0.495 | 0.11632 | 23.50 | 0.267 | 0.723 | 0.0554 | 0.1125 | 78.1 | 5.4 | 2 | 2.6 | 5.3 |
| 42129803200 | 0 | -79.74556433 | 40.3588067 | 0.491 | 0.12084 | 24.62 | 0.254 | 0.728 | 0.1831 | 0 | 83.1 | 3.7 | 5.4 | 4.1 | 0 |
| 42129803301 | 1 | -79.7654123 | 40.33609352 | 0.581 | 0.06148 | 10.58 | 0.460 | 0.702 | 0.0426 | 0.2883 | 80.8 | 1.2 | 2.7 | 2.8 | 1.9 |
| 42129803302 | 1 | -79.76651087 | 40.3090152 | 0.369 | 0.09513 | 25.78 | 0.183 | 0.555 | 0.1264 | 0.2924 | 77.6 | 3.8 | 4.2 | 5 | 2.8 |
| 42129805100 | 0 | -79.76379277 | 40.26418676 | 0.570 | 0.11849 | 20.77 | 0.338 | 0.803 | 0.6366 | 0.2096 | 82 | 13.9 | 6.6 | 10 | 1.3 |
| 42129805800 | 0 | -79.84769647 | 40.16590724 | 0.468 | 0.12038 | 25.73 | 0.232 | 0.704 | 0.2044 | 0.1521 | 83 | 7.8 | 2.9 | 6 | 7.1 |

**Table S3.** Data dictionary for the variables in Table S2

| **Variable** | **Definition** |
| --- | --- |
| TRACT | Census tract FIPS code |
| Indicator | 0 - Non-sample, 1 - Sample |
| CX1 | Centroid longitude |
| CX2 | Centroid latitude |
| Small area estimates | Small area estimate for proportion of ever smokers |
| SE | Standard error |
| %CV | Coefficient of variation |
| Lower | Lower 95% confidence limit |
| Upper | Upper 95% confidence limit |
| RPL_THEME1 | Socioeconomic theme summary (Social Vulnerability Index or SVI) |
| RPL_THEME3 | Minority Status & Language theme summary (SVI) |
| Adult_Percent | Percentage of population of age 18 years or above |
| Poverty_Percent | Percentage of population in poverty |
| Unemployed_Percent | Percentage of population unemployed |
| NoHighSchoolDiploma_Percent | Percentage of population with no high school diploma |
| Minority_Percent | Percentage of minority population |

**Table S4.** Tracts removed from small area analysis

| **TRACT** | **Indicator** | **CX1** | **CX2** | **RPL_THEME1** | **RPL_THEME3** | **Adult_Percent** | **Poverty_Percent** | **Unemployed_Percent** | **NoHighSchoolDiploma_Percent** | **Minority_Percent** |
| --- | --- | --- | --- | --- | --- | --- | --- | --- | --- | --- |
| 42003980900 | 1 | -80.0419 | 40.46659 | -999 | 0.4227 | 65.4 | 50 | 0 | 0 | 0 |
| 42003980100 | 0 | -79.9145 | 40.48122 | -999 | 0 | 100 | -999 | -999 | -999 | 0 |
| 42003980300 | 0 | -79.9057 | 40.43255 | -999 | -999 | 100 | -999 | -999 | -999 | 0 |
| 42003980400 | 0 | -80.02 | 40.48194 | -999 | -999 | 100 | 0 | -999 | 0 | 0 |
| 42003980600 | 0 | -80.028 | 40.45199 | -999 | 0 | 100 | 0 | 0 | 0 | 0 |
| 42003980700 | 0 | -80.0118 | 40.43764 | 0 | 0 | 100 | -999 | -999 | -999 | 0 |
| 42003980800 | 0 | -79.9654 | 40.43257 | -999 | -999 | 100 | -999 | -999 | 18.7 | 47.2 |
| 42003981000 | 0 | -79.9489 | 40.4731 | -999 | -999 | 100 | -999 | -999 | -999 | 0 |
| 42003981100 | 0 | -79.9063 | 40.44204 | -999 | 0 | 100 | 0 | 0 | 0 | 0 |
| 42003981200 | 0 | -80.0108 | 40.44646 | -999 | -999 | 100 | -999 | -999 | -999 | 0 |
